# A regional assessment of cumulative impact mapping on Mediterranean coralligenous outcrops

**DOI:** 10.1038/s41598-018-20297-1

**Published:** 2018-01-29

**Authors:** S. Bevilacqua, G. Guarnieri, G. Farella, A. Terlizzi, S. Fraschetti

**Affiliations:** 1 Department of Biological and Environmental Sciences and Technologies, University of Salento, 73100 Lecce, Italy; 2 CNR-ISMAR – Institute of Marine Sciences, Venezia, Italy; 3 Department of Life Sciences, University of Trieste, 34127 Trieste, Italy; 4 Stazione Zoologica Anton Dohrn, 80121 Napoli, Italy; 5 CoNISMa, Piazzale Flaminio 9, 00196 Roma, Italy

## Abstract

In the last decade, the ‘Cumulative Pressure and Impact Assessment’ (CPIA) approach emerged as a tool to map expected impacts on marine ecosystems. However, CPIA assumes a linear response of ecosystems to increasing level of cumulative pressure weighting sensitivity to different anthropogenic pressures through expert judgement. We applied CPIA to Mediterranean coralligenous outcrops over 1000 km of the Italian coastline. Extensive field surveys were conducted to assess the actual condition of coralligenous assemblages at varying levels of human pressure. As pressure increased, a clear shift from bioconstructors to turf-dominated assemblages was found. The linear model originally assumed for CPIA did not fit the actual relationship between expected cumulative impact versus assemblage degradation. A log-log model, instead, best fitted the data and predicted a different map of cumulative impact in the study area able to appreciate the whole range of impact scenarios. Hence, the relative importance of different drivers in explaining the observed pattern of degradation was not aligned with weights from the expert opinion. Such findings stress the need for more incisive efforts to collect empirical evidence on ecosystem-specific responses to human pressure in order to refine CPIA predictions.

## Introduction

Worldwide, marine coastal systems are threatened by increasing human pressures often acting simultaneously^1^. Ecological research has documented the impact of individual stressors on species, habitats and ecosystems. Studies have shown that sedimentation, nutrient enrichment, pollution, resource exploitation, presence of non-indigenous species, habitat destruction and fragmentation can alter ecosystem functioning at varying scales in time and space, changing the number and composition of species and their relative abundances through direct and indirect effects^2,3^.

The need for a deeper understanding of the effects of multiple stressors on ecosystems was highlighted about twenty years ago^4^ and is still considered one of the most challenging questions for ecosystem-based management^5^. In this framework, environmental impact assessments have attempted to move from considering single-source of impact towards more comprehensive approaches investigating ecological responses to multiple interacting human disturbances^6^. Mesocosms^7^, manipulative^8^ or correlative field experiments^9^, and modelling^10^ have been used to quantify the effects of multiple stressors on marine biodiversity. More recently, the recognition that human activities and their potential impacts are spatially explicit has led to the development of the cumulative pressure and impact assessment (CPIA) approach^6^, which focuses on mapping the distribution of human pressures and expected impact on marine ecosystem. However, despite its application to different environmental contexts worldwide (e.g.^1,11,12^), the CPIA approach still relies on the assumptions that the effects of pressures are fully additive and that cumulative impacts increase linearly at increasing pressures^13^. This could be not retained to changing environmental settings, strongly affecting the reliability of the ensuing impact estimates^14^. A further issue in CPIA concerns the use of scores based on expert judgement to weight the potential effects of anthropogenic pressures on different ecosystems. Although scores from expert opinion could represent useful proxies of ecosystems sensitivity, comparisons with quantitative assessments raised doubts on their appropriateness in weighting the actual effects of human pressures on marine systems^15^. Indeed, a note of caution on the estimated cumulative impacts from CPIA is intrinsic to the approach^6^, unless accompanied with careful ground-truthing^16^, and information on relationships between expected impact and the actual condition of habitats and assemblages.

The Mediterranean Sea is largely affected by multiple stressors leading to a serious loss of marine biodiversity and the degradation of ecosystem functioning^17^. Estimates of cumulative impacts at basin scale (i.e.,^12^) highlighted that 60–99% of the territorial waters of EU member states are subject to high impact. Coralligenous outcrops are mainly produced by the accumulation of calcareous algae and invertebrates. They characterize at least the 30% of the coasts of the basin^18^ and represent the most important Mediterranean marine habitat, in terms of biodiversity and productivity, after *Posidonia oceanica* meadows^19^. Several studies addressed the effects of single sources of impact (e.g., waste waters, fishing, sedimentation, thermal anomalies) on this habitat at local scale^9,20^. However, there is an absence of large-scale quantitative assessments of the effects of different combinations of human pressures, impairing the understanding of the processes leading to structural and functional shifts in this system^21^.

Here, we applied the CPIA framework on Mediterranean coralligenous outcrops over 1000 km of coast (Apulia, SE Italy), combining, for the first time, detailed information on human pressures and maps of habitat distribution with an extensive field survey to directly assess the state of coralligenous assemblages at varying levels of human pressure. The aim of this study is to understand the relationship between human pressures and this priority habitat at regional scale, addressing two of the most relevant sources of uncertainty in CPIA: (i) the assumption of a linear relationship between the estimated cumulative impact and the actual condition of the investigated assemblages, and (ii) the relation between pressure weights from expert opinion and actual correlations among pressures and assemblage responses. The outcomes are expected to shed light on factors affecting the effectiveness of the CPIA approach to provide reliable estimates of potential effects of multiple anthropogenic pressures, contributing to inform spatial management of cumulative impacts on marine systems.

## Results

### Cumulative pressure level on coralligenous outcrops

**Figure 1:**
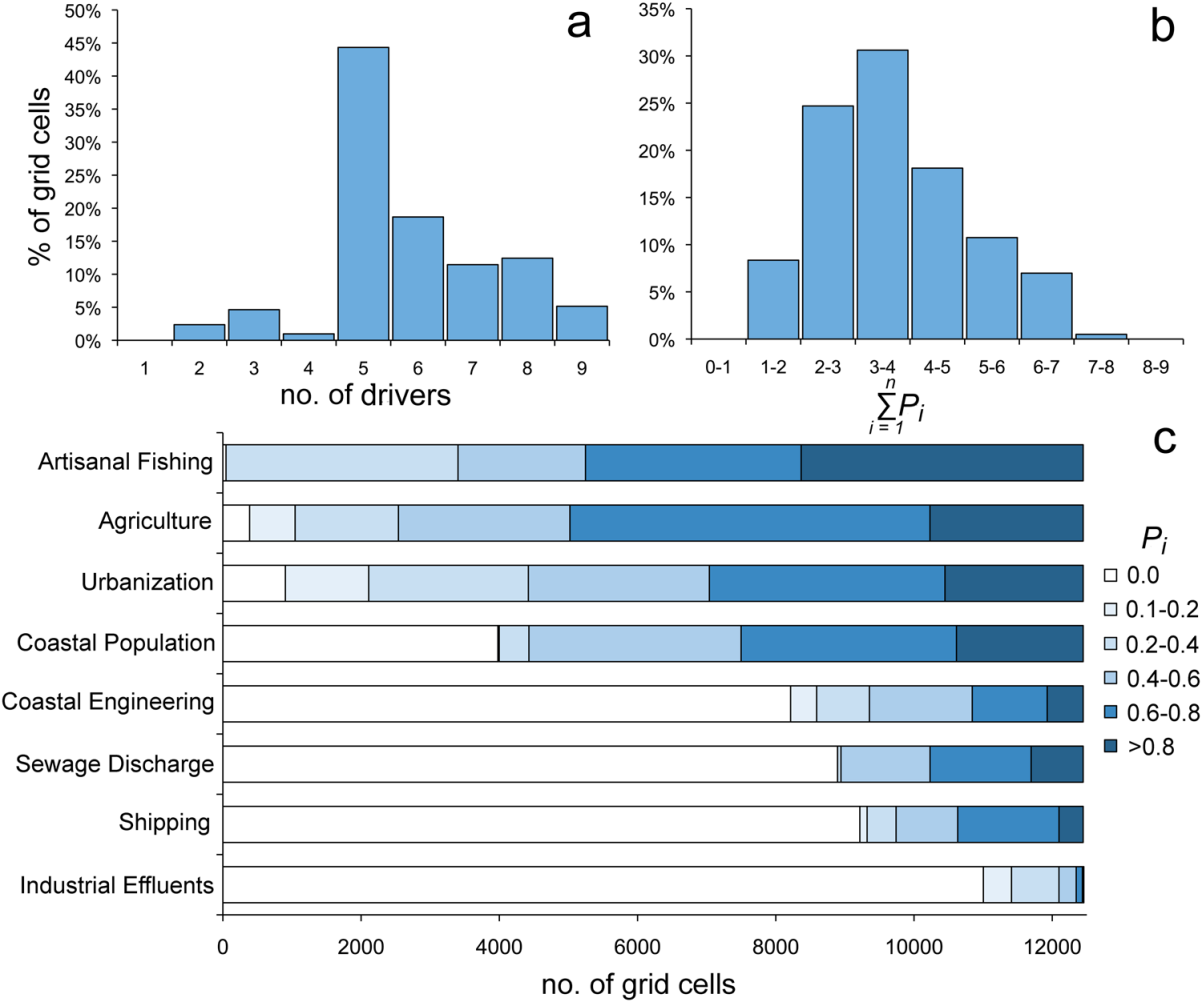
(**a**) % of grid cells per number of drivers. (**b**) % of grid cells exposed to different levels of cumulative pressure (${\sum }_{i=1}^{n}{P}_{i}$, where *P*_*i*_ is the value of the pressure associated to the driver i). (**c**) Pressure of single drivers per number of grid cells (Acidification was excluded due to the fact that it acts uniformly over the region).

A total of 12,447 cells of the grid were characterized by coralligenous outcrops on the sea bottom within 30 m depth (about 498 km^2^). No cells were completely unaffected by anthropogenic drivers, and more than 40% of cells resulted exposed at least to 5 of them (Fig. 1a). Less than 10% of cells seemed to receive the potential influence of fewer (<4) drivers, whereas about half of coralligenous outcrops were potentially influenced by a larger number of drivers ≥ 6 (Fig. 1a). However, most cells (>60%) showed relatively low levels of cumulative pressure (i.e., ${\sum }_{i=1}^{n}{P}_{i} < 4$), whereas the remaining cells (about 30%) exhibited medium levels (4–6) and only ∼8% were exposed to high (>6) levels of cumulative pressure (Fig. 1b). The most frequent drivers, irrespective of their pressure level, were *Artisanal Fisheries* (>99% of cells), *Agriculture* (97%), and *Urbanization* (93%), whereas the least frequent ones were *Industrial Effluents* (12%), *Shipping* (26%), *Sewage Discharge* (29%), and *Coastal Engeneering* (34%); the most frequent drivers were also those acting in most cells with medium to high levels of pressure (Fig. 1c).

### State of the assemblages and relationships with anthropogenic pressures

**Figure 2:**
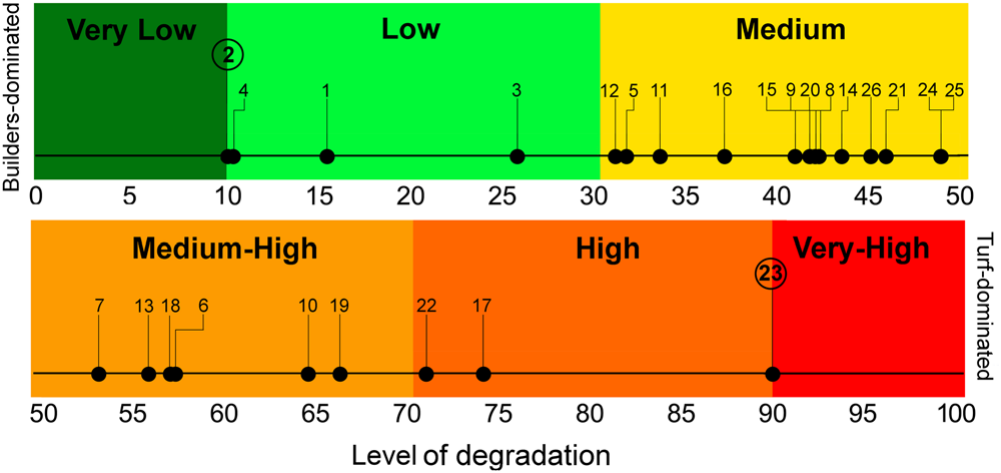
Condition of assemblages from PCoA analysis in the 26 investigated sites (see Fig. S9). Values of sampled sites along axis 1 of PCoA from the best (Site 2) and the worse (Site 23) recorded conditions were rescaled to vary between 10 and 90. Positions of Site 2 and 23 were assumed to mark the limit between very low-low and between high-very high degradation, respectively. Thresholds from very low to very high were set analogously to Halpern *et al*.^6^ and corresponded to a gradient of increasing degradation of coralligenous outcrops from a condition in which calcified algae and invertebrate builders were dominant towards a turf-dominated condition.

The two axes of the PCoA ordination plot explained the 99% of the total variation in assemblage structure (see Figure S9 in Supplementary material) based on four main morpho-functional components of coralligenous outcrops, namely *Calcified Algae*, *Invertebrates*, *Erect Macroalgae*, *Turf Algae* (see Appendix S2). The 84% of total variation among sites was explained by the PCoA axis 1, which showed a clear shift from sites with assemblages characterized by the dominance of turf-forming algae (left side) to sites where typical bioconstructors of coralligenous outcrops (i.e., encrusting and erect calcified algae and invertebrates) were dominant (right side) (Figure S9). For each sampled site, the level of degradation derived from PCoA axis 1 was reported in Fig. 2. Sites 1–4, which were located within MPAs and in most cases far from the coast in areas featured by low levels of human pressure, showed a low level of degradation; the majority of sites exhibited medium or medium-high degradation, whereas sites 17, 22, 23, exhibited high to very high level of human pressure of these areas (Fig. 2).

**Figure 3:**
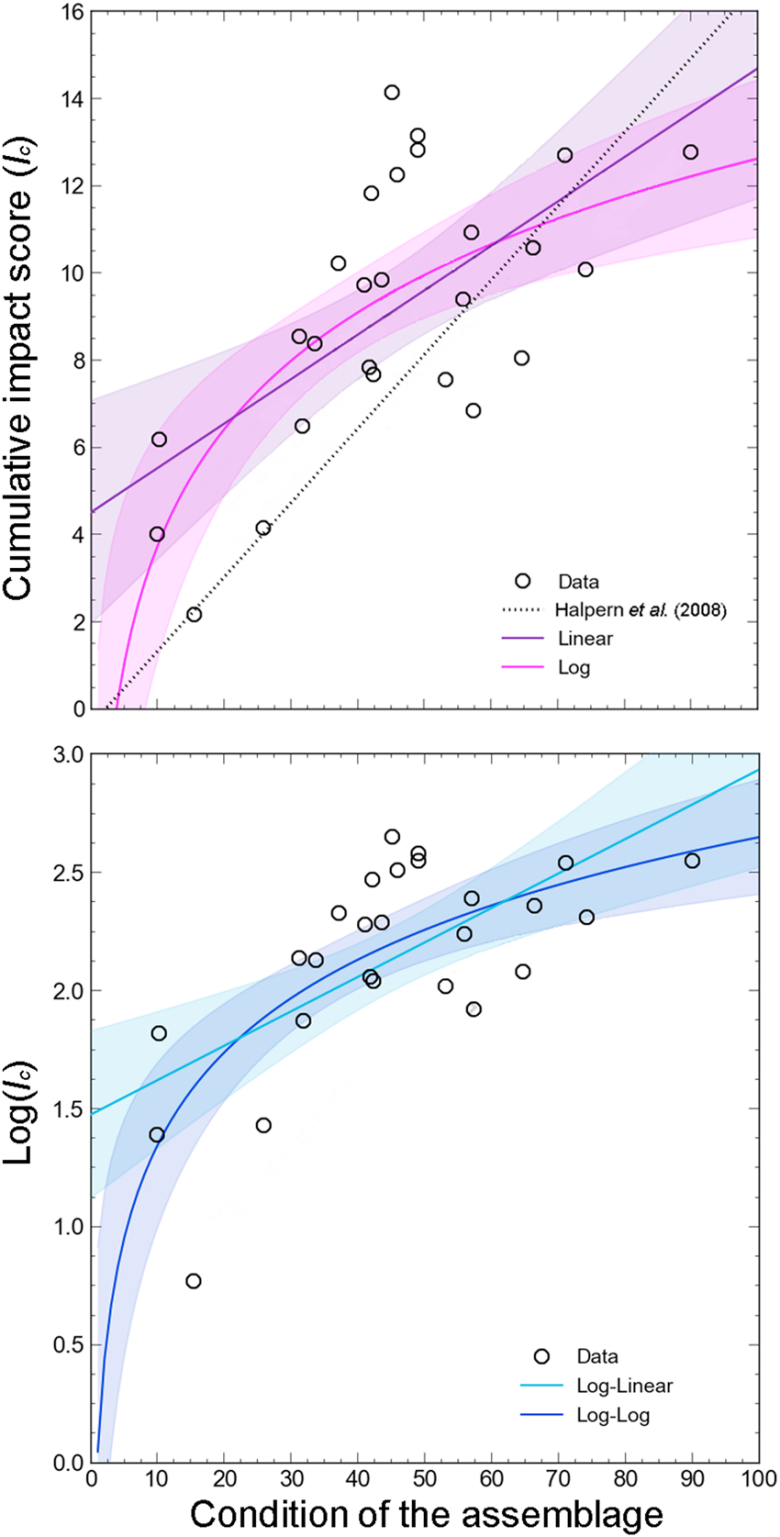
Linear/natural logarithmic (upper plot) and log-linear/log-log (lower plot) models of cumulative impact score (*I*_*c*_) against the condition of coralligenous assemblages. The linear relationships provided by Halpern *et al*.^6^ was also showed (upper plot). Shaded areas around regression lines represented the 95%CI.

Plot of the residuals of actual data on coralligeous outcrops against the expected conditions calculated following the linear relationship identified by Halpern *et al*.^6^, indicated that such linear model did not explain adequately the relationship between *I*_*c*_ and the true condition of coralligenous assemblages (Figure S10 in Supplementary material). The Wald-Wolfowitz runs test returned a probability of *P* = 0.029 (number of runs = 8), indicating that the observed pattern in the residuals is non-random, and therefore the specific linear relationship (*I*_*c*_ = 0.1762 × [level of system degradation] − 0.3381) found by Halpern *et al*.^6^ is unlikely to be robust also for coralligenous outcrops (Fig. 3). Runs test indicated that all models (linear, logarithmic, log-linear, and log-log) used to fit actual data on the relationship between *I*_*c*_ versus assemblage conditions were not unlikely (Table 1). Regression analysis and AICc indicated that the best fit of data was achieved by using a log-log model (Table 1, Fig. 3). Thresholds in *I*_*c*_ strongly varied among different models, although all models led to increase the limits for categories that define less impacted conditions (Table 1).

**Table 1:** Results of regression analysis of cumulative impact score (*I*_*c*_) against the state of coralligenous outcrops using different models.

Regression analysis						
Model	*R* ^2^	SE	Significance level	AICc	Run test	
					*P* (%)	no. of runs
Linear	0.40	2.41	***	45.90	57.93	15
Log	0.47	2.28	***	42.93	59.19	15
Log-Linear	0.42	0.33	***	−57.02	57.93	15
Log-Log	0.51	0.31	***	−61.50	43.23	14
**Ranks of cumulative impact score**						
		**Thresholds**				
**Degradation level**		**Halpern** *et al.* ^6^	**Linear**	**Log**	**Log-Linear**	**Log-log**
10 (Low)		1.40	5.55	3.78	5.07	3.86
30 (Medium)		4.95	7.58	8.00	6.79	7.19
50 (Medium-High)		8.47	9.62	9.97	9.10	9.59
70 (High)		12.00	11.66	11.26	12.18	11.60
90 (Very high)		15.52	13.69	12.23	16.31	13.37

Results of AICc and run tests were also reported. SE = standard error of regression. ***P < 0.001. For each model, the corresponding thresholds for ranks of cumulative impact score were provided. Thresholds from Halpern *et al*.^6^ were also reported.

Distance-based multivariate multiple regression (DISTLM) showed that the 53.2%, of total variability among sites was explained considering the full set of drivers. Marginal tests on single drivers showed that correlation was significant only for *Coastal Population*, *Shipping*, *Sewage Discharge*, and *Coastal Engineering*, which in turn, also mostly contributed to the explained variation in coralligenous assemblages among sites (Table 2). Weights assigned to drivers based on expert opinion only partially matched the contribution to explain the observed patterns in coralligenous assemblages. Drivers with high weight (i.e., considered as having a great impact) from expert opinion had a weak, not significant, relationship with the actual condition of the assemblages, except for *Coastal Population* (Table 2). In contrast, drivers with lower weights (<2) showed a significant and relatively high correlation with multivariate patterns of assemblages (Table 2).

**Table 2:** Results of marginal tests and contribution of each driver to explain the multivariate pattern of variation along the gradient of degradation of coralligenous outcrops from sampled sites.

Driver	Pseudo-*F*	*P*	Explained variation	*w*_*i*_ from Halpern *et al*.^36^
*Coastal Population*	11.85	**0.001**	0.33	2.5
*Artisanal Fisheries*	0.52	0.553	0.02	2.3
*Urbanization*	1.91	0.176	0.07	2.2
*Agriculture*	2.37	0.120	0.09	2.2
*Sewage Discharge*	6.12	**0.017**	0.20	2.1
*Coastal Engineering*	3.39	**0.050**	0.12	1.9
*Industrial Effluents*	1.55	0.213	0.06	1.6
*Shipping*	9.56	**0.002**	0.28	1.4

Significant results were given in bold. For each driver, weights (*w*_*i*_) based on expert opinion from Halpern *et al*.^36^ were also provided.

### Regional map of expected cumulative impact

**Figure 4:**
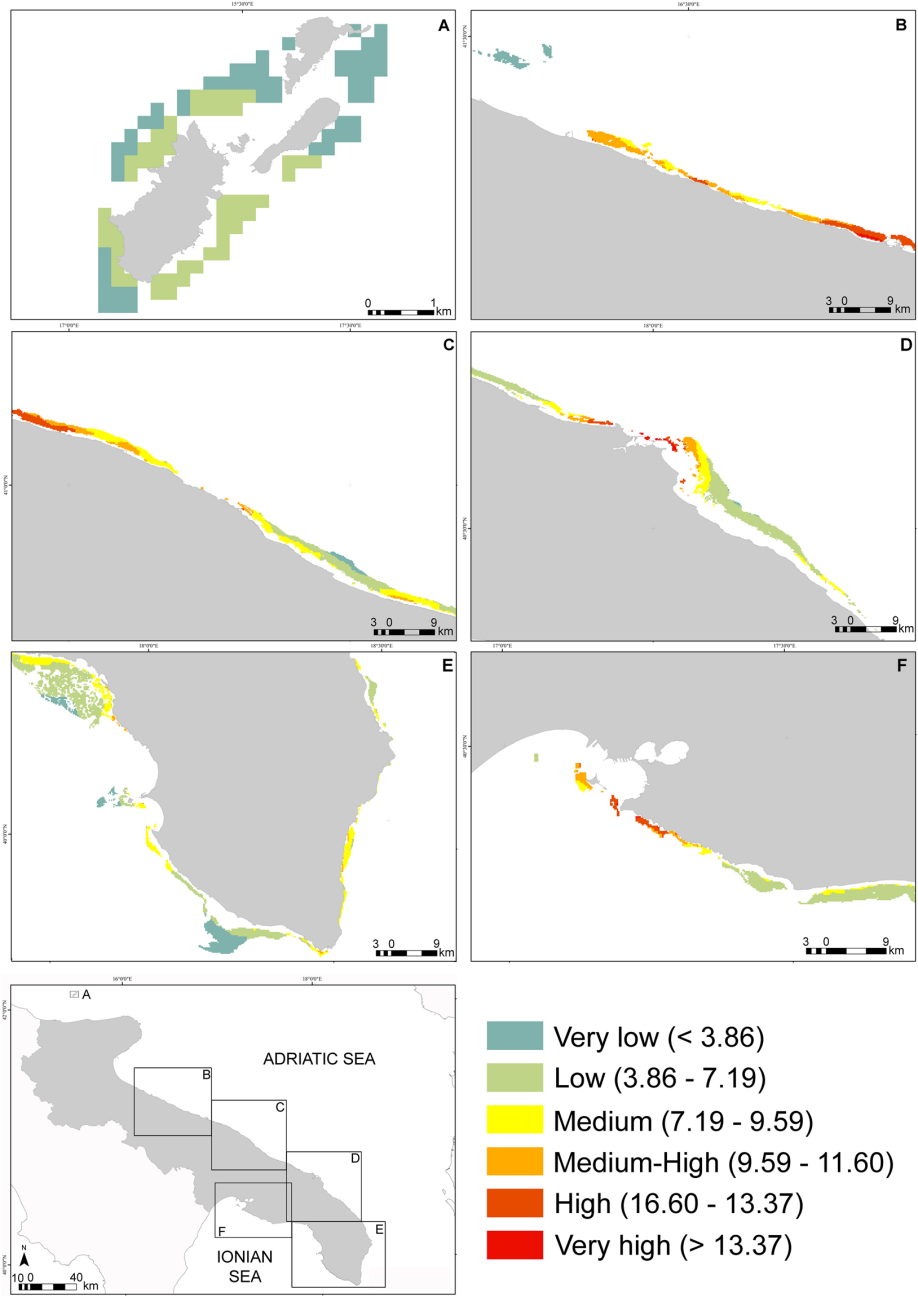
Regional map (WGS84) of cumulative impact score (*I*_*c*_) to coralligenous assemblages calculated following the best fitting model (log-log). The whole extent of coralligenous outcrops within 30 m depth at a regional scale was split into six sectors (**a**–**f**) to help displaying the spatial distribution of *I*_*c*_. All coloured polygons in the map represent the areas characterized by the presence of coralligenous outcrops, whereas different colours indicated different levels of the expected cumulative impact on the outcrops. Limits of *I*_*c*_ defining different ranks of expected impact, from very low to very high, were reported in brackets. Maps were created using the ArcGIS® 10.1 software by ESRI (Environmental Systems Resource Institute, http://www.esri.com).

The regional map of cumulative impact of coralligenous outcrops was finally produced based on the new thresholds calculated from the log-log relationship between *I*_*c*_ and the condition of coralligenous outcrops (Fig. 4). The highest and widespread expected cumulative impact was recorded in sectors B and C, which are among the most populated and urbanized stretches of coast in the region. Spots of high or very high cumulative impact, corresponding to large coastal urban settlements, characterized sectors D and F. Sector E (a poorly urbanized stretch of coast along a huge terrestrial reserve) and A (a protected archipelago 12 nautic miles off the coast) appeared as the less impacted areas.

**Figure 5:**
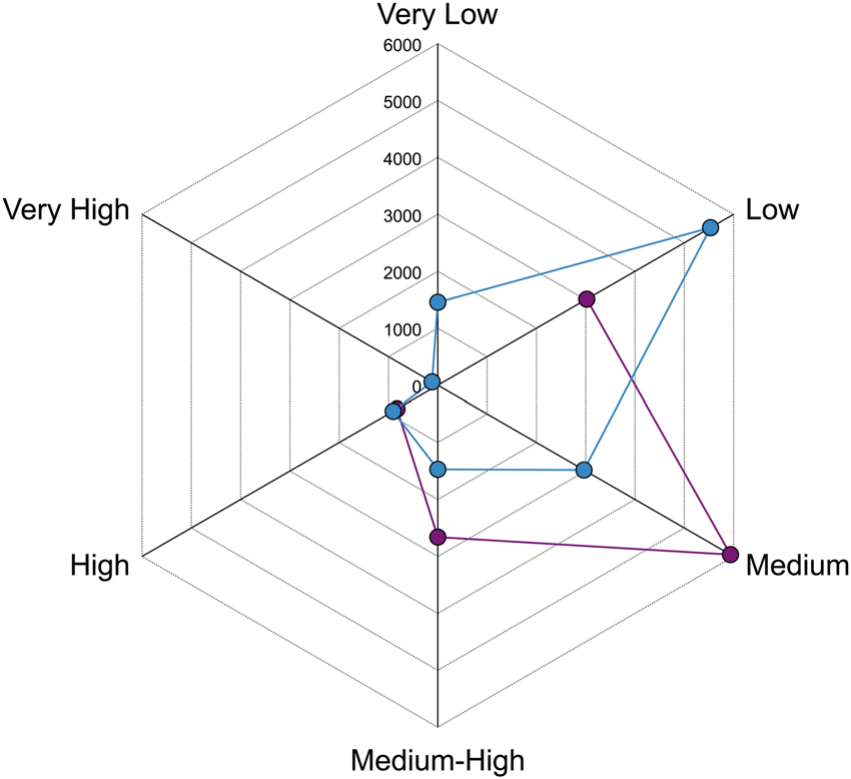
Spider plot of the number of grid cells assigned to the different classes of *I*_***c***_ following thresholds based on the linear model provided by Halpern *et al*.^6^, and the new thresholds based on the log-log model from actual data on coralligenous outcrops (respectively reported in violet and blue). Each of the six axes referred to each class of *I*_***c***_.

Maps of regional cumulative impact on coralligenous outcrops changed substantially whether using thresholds from Halpern *et al*.^6^ or the new thresholds obtained from the best fitting (log-log) model based on actual data (Fig. 5). The classic ranking led to assign all cells to 4 categories, i.e. low, medium, medium-high, high, with cumulative impact classified as medium in about half of cells. The new ranking, instead, identified also a small portion of very highly impacted cells and about 10% of cells with very low impact; most of cells were classified as subjected to a low impact.

## Discussion

The use of refined data on the distribution and intensity of human pressures coupled with a habitat-specific calibration of thresholds in impact scores provided a more realistic picture of the severity of cumulative impact on coralligeous outcrops. The use of thresholds from Halpern *et al*.^6^ led most of coralligenous in the region to be classified as medium-impacted, with no areas of very low or very high impact. In contrast, the use of thresholds from the best fitting model based on actual data allowed us to discriminate the whole range of impact classes and, although depicting a general condition of low impact similarly to other cumulative impact assessments on the same habitat in the Mediterranean Sea (e.g.^22^), highlighted also the presence of very highly impacted cells deserving management priority. Interestingly, all sites included within MPAs exhibited low degradation, emphasising the critical role of protection in preventing human-driven shifts towards undesirable ecological conditions^23^ and increasing the resilience of assemblages^24^. This is a very strong argument supporting the use of MPAs as reference sites in large scale monitoring programs, such as those involved in the Marine Strategy Framework Directive (MSFD^25^), since the knowledge of baselines is lacking for most of marine assemblages and habitats and sound reference conditions represents a fundamental step in any quality status assessment^26^.

Our findings show that, irrespective of the relevant geographic extent (about 1000 kms) of the study area, distinct patterns of coralligenous degradation were associated to different levels of human pressure over and beyond spatial variability and changes in environmental conditions. At increasing level of pressures, a clear shift from assemblages featured by bioconstructors to assemblages dominated by turf-forming algae was detected. A complex suite of interactions emerged from our analyses on multiple pressures, indicating a critical role of coastal population, shipping, sewage discharge, and coastal engineering in driving the observed patterns of degradation. In most cases, coralligenous outcrops were subjected to a medium-high number of different drivers. Artisanal fishery and land based pollution (e.g., agriculture and urbanization) were the drivers acting at broad scale across the region, although accounting for a minor contribution to the observed patterns of degradation. Interestingly, sensitivity weights assigned by expert opinion to agriculture and artisanal fisheries were higher than those assigned to drivers strongly correlated with degraded conditions, suggesting that calibrations based on actual data are strictly necessary to adapt general sensitivity weights to regional or local contexts. This, in turn, could provide an objective basis to guide management policies in prioritizing actions on those anthropogenic drivers that could cause major cumulative impacts. In most cases, current knowledge on the relative importance of different drivers for ecosystem shifts and all potential synergies (or antagonism) is still incomplete. We can just assume that the effects of multiple stressors are additive. Stressors can nevertheless interact in complex ways, and non-additive effects have been demonstrated to be common in nature^27^. As an example, nutrient enrichment is recognized as a global problem associated with a range of human activities, with non-additive interactions with other stressors^28^ such as, for instance, physical damage from destructive fishing practices (e.g.^8^). Disentangling the combined effects of multiple stressors is, more than ever, necessary for a successful management of marine ecosystems^29^, and structured experimental designs with factorial combinations of stressors can provide invaluable insights in this perspective.

Mapping the distribution of human pressures and the ensuing cumulative effects is critical to inform the management of offshore and coastal zones^30^, as also recognized by recent commitments in Europe (e.g., MSFD) and elsewhere^31^. In this view, the cumulative impact score proposed by Halpern *et al*.^6^ has rapidly developed into a global standard and adapted to a variety of scenarios and scales, providing a general indication of the ecological status of several areas in the ocean^11^. The main concern is that aggregating the relative contribution of different pressures into a single index may not reflect important interactions that exist among individual sources of disturbance, environmental conditions and ecological responses, which likely represents a major cause of uncertainty for CPIA predictions^32,33^. A major issue relies on the fact that a given anthropogenic driver may exert multiple types of pressure. Since it is not the driver *per se*, but rather the associated pressures that ultimately relate to the ecological response, weighting drivers to estimate the expected impact in CPIA could be misleading, unless we assume that each pressure correspond to a specific driver, which is often not the case in the real world. The erroneous interchangeability of the concepts associated to the terms ‘driver’ and ‘pressure’ in the original method further contributed to generate confusion in the application of CPIA, although this ambiguity has been amended in recent reformulation of the approach (see^1,34,35^).

Congruence of cumulative impact scores is still a central question in CPIA, since the power to discriminate among expected levels of impact can change substantially depending on data resolution, thresholds of impact scores and weights assigned to anthropogenic drivers^33^. As recently discussed by Korpinen and Andersen^13^ in a global review on CPIA studies, in most works the effects of different pressures in order to calculate cumulative impact scores were weighted based on expert judgement (e.g.^36^). However, the understanding of potential effects of human pressures on different ecosystems is far from being exhaustive, thus limiting the possibility of comparisons between empirical evidence and expert opinion^12,37^. In addition, despite several studies used global weights in regional CPIA assessments (e.g.^12,38^), others have stressed the importance to calibrate CPIA to the specific region of interest (e.g.^15,39,40^). We found a substantial mismatch between sensitivity to pressures attributed by experts and correlations of pressures with the actual conditions of coralligenous outcrops. It could be argued that, irrespective of associated sensitivity weights, the most widespread and intense pressures were likely to be more correlated to changes in assemblage condition than those with lower intensity and reduced spatial coverage. Therefore, the observed mismatch could not be univocally interpreted as a departure of weight scores from the true habitat sensitivity to different pressures. In our case, however, most of pressures with limited spatial extension and intensity (i.e., *Sewage discharge*, *Coastal Engineering*, *Shipping*) were among the main drivers of the observed patterns in assemblage condition despite their relatively low weight scores (see Fig. 1 and Table 2), suggesting that, over and beyond pressure intensity and distribution, the development of context-specific weights might be more appropriate than the use of global weights for regional CPIA assessements^14^.

The level of estimation of expected impact from human pressures may severely affect the calculation of the cumulative impact score, which in turn, could mislead management actions. To date, very few studies concerned the validation of CPIA predictions by contrasting actual versus expected conditions of marine systems. Andersen *et al*.^16^ found a substantial alignment of expected impacts from CPIA with ecosystem condition, working at basin scale in the Baltic Sea. However, basin-scale CPIA approaches are likely to be adequate for sub-basin or regional management only if available data on ecosystems and threats insisting on the area are accurate enough to discriminate local conditions^41^. Downscaling expected impact from large to regional or local scale may be even more problematic in particular areas, such as the Maditerranean Sea^12^, due to the heterogeneity of habitats and distribution of threats^15^. Also, ground-truthing CPIA predictions may result in relatively weak relationships between expected impacts and actual conditions of ecosystems if the range of pressure level is small^41^, which could be often the case at a local scale.

Despite the fact that several issues may contribute to the uncertainty of predictions^33,35^, the CPIA approach is increasingly applied without substantial modifications to its original formulation. This is particularly significant considering the assumption of a linear relationship between pressure level and expected impact^32^, which is at the core of impact score assignments. We demonstrated that, at least for coralligenous outcrops, the linear model and derived thresholds in *I*_*c*_ provided by Halpern *et al*.^6^ failed to explain adequately the relationship between cumulative impacts and the condition of the system, casting doubts on their general application to a variety of ecosystems and geographic contexts. In our case, a non-linear, and specifically a log-log model, described better the relationship between *I*_*c*_ vs. the condition of the system. This pattern of pressure-state relationship appears rather plausible with respect to linearity of response. It reflects the ‘cliff’ paradigm^42,43^, in which natural ecosystems are viewed as resilient entities, able to absorb anthropogenic disturbance, at least until a certain level. Beyond this level the risk of ecosystem collapse is high, and if occurring, difficult to return to the original condition^44^. Analogously, our log-log empirical relation between coralligenous response *versus* increasing expected impact implies that the system may initially resist to pressure so that, a relatively large rise in cumulative pressure could have only limited effects. Once pressure intensity further increases, effects become more and more evident and, above certain levels, the system could rapidly deteriorate even as a consequence of small increments in cumulative pressure. Due to widespread evidence sustaining non-linearity of response in real-world ecosystems (e.g.^33,45^), generalizations on the pressure-state model to use in CPIA appears unfeasible. Such findings reinforce the idea that the use of weights (*w*_*i*_) allows a calibration of the cumulative impact score (*I*_*c*_) only in terms of the sensitivity of different systems to different pressures^13^, emphasising the need to integrate calculation of *I*_*c*_ to account also for ecosystem-specific responses to increasing pressure level.

Maintaining biodiversity and achieving a Good Environmental Status (GES) of marine environments through an ecosystem-based approach to the management of human activities and a sustainable use of marine goods and services is the ultimate aim of the European MSFD^44^. Hence, a specific requirement of the MSFD is to consider the cumulative synergistic effects of human pressure in the assessment of GES^34^. Due to the dimension of the challenge, reliable tools for a rapid assessment of the condition of ecosystems and the detection of early warning signals of potential regime shifts are strongly advocated^46^. Our results confirm the potential of CPIA as a profitable framework to model the expected conditions of marine systems based on the distribution of human pressures, representing a cost-effective approach for marittime spatial planning. What is urgently needed is a more incisive effort to synthetize available information and fill existing gaps in linking human pressures and the response of ecosystems, in order to improve the effectiveness of CPIA in setting priority areas for conservation, mitigation, and restoration strategies. This study, by providing new information on patterns of degradation and sensitivity to pressure of a priority habitat, such as coralligenous outcrops, is a first step in this direction and represents a concrete effort to improve the effectiveness of CPIA that can be extended to other habitats in the Mediterranean and elsewhere.

## Methods

### Cumulative pressure and impact assessment on coralligenous outcrops

A continuous habitat map (1:25000) based on georeferenced data on the occurrence of coralligenous outcrops up to 30 m depth along the Apulian coasts was obtained from mapping activities (http://www.sit.puglia.it/portal/portale_rete_ecologica/biomap), which combined high-resolution morphobathymetric data (Multibeam echosounder), sismostratigraphic profiles (Chirp sonar and sub-bottom profiler), and acoustic seabed photogrammetry (using Side Scan Sonar) at regional scale.

CPIA on coralligenous outcrops was conducted following the framework of Halpern *et al*.^6^. As first, we considered all threats to marine ecosystems from the comprehensive list provided in Halpern *et al*.^36^. Fourteen threats, out of a total of 35 in the list, were not considered in the analysis since they were absent from (i) the whole region (*Ocean mining*, *Offshore development*), (ii) the areas where the investigated habitat is distributed (*Demersal, destructive* and *non-destructive fishing*, *Pelagic-high bycatch fishing*, *Freshwater input*), (iii) the depth range characterizing the investigated outcrops (*Sea temperature increase*, *Sea level rise*, *Ozone/UV*, *Harmful algal blooms*, *Hypoxia*), or negligible (*Atmospheric pollution*, *Species invasion*). Nine additional threats (*Benthic Structures*, *Ecoturism*, *Diseases*, *Aquarium fishing*, *Illegal fishing*, *Artisanal, non-destructive fishing*, *Recreational fishing*, *Sediment input*, *Nutrient input*) were also excluded since no data were available (see Table S1 in Supplementary material for further details). A total of 12 threats were retained and spatial data on related anthropogenic drivers (i.e., *Sewage discharge*, *Industrial Effluents*, *Agriculture*, *Urbanization*, *Coastal Engineering*, *Coastal Erosion*, *Coastal Population*, *Aquaculture*, *Artisanal Fisheries*, *Ocean Acidification*, *Shipping*, *Commercial Activity*) were collected and mapped (Table S1). The intensity of pressures associated to the 12 drivers was quantified in terms of: (1) population equivalent for *Sewage discharge*, (2) land cover of industrial areas within 300 m from the coast for *Industrial Effluents*, (3) land cover of agricultural areas within 1 km from the coast for *Agriculture*, (4) land cover of urban areas within 1 km from the coast for *Urbanization*, (5) size of coastal structures for *Coastal Engineering*, (6) landward beach displacement for *Coastal Erosion*, (7) population size and density for *Coastal Population*, (8) surface of farmed areas for *Aquaculture*, (9) number of vessels per size categories for *Artisanal Fisheries*, (10) aragonite saturation state for *Ocean Acidification*, (11) total tonnage of shipped goods for *Shipping*, and (12) number of ship tracks per cell for *Commercial Activity*. The overlay analysis between the spatial distribution of the investigated habitat and the area of influence of *Coastal Erosion*, *Aquaculture*, and *Commercial Activity* allowed to exclude any potential interference with coralligenous outcrops within 30 m depth. All modelled spatial data layers concerning the origin and intensity of the remaining pressures were integrated and used in the CPIA analysis and maps. A detailed description of threats and related anthropogenic drivers considered in the analysis is provided in the Supplementary material.

All layers on anthropogenic pressures and coralligenous distribution were mapped through ArcGIS® 10.1 software by ESRI (Environmental Systems Resource Institute, http://www.esri.com/software/arcgis) on a 200 × 200 m (0.04 km^2^) pixel grid. For each driver, the value of the associated pressure assigned to the cell was that of the raster pixel falling at its center. Negative exponential models were used to estimate the distance decay from 100% until 0% in the intensity of pressures on a 200 m-distance raster matrix. A further theoretical decrease in the intensity of pressures equal to 10% each 10-m depth increment was applied to account for reduction of potential effects of pressures due to bathymetry^22^. Full details on drivers, associated pressures, and data treatments, along with distribution maps at regional scale, are provided in the Supplementary material. All values were normalized applying a log(X+1) transformation and rescaled in the range 0 to 1^12^.

According to Halpern *et al*.^6^ and subsequent formulations^1,34^, the cumulative impact score on coralligenous outcrops (*I*_*c*_) was calculated following the equation (1):

1 $${I}_{c}=\sum _{i=1}^{n}{P}_{i}{E}_{c}{w}_{i}$$

where *n* is the number of drivers within the examined grid cell, *P*_*i*_ is the normalized value of the pressure associated to the anthropogenic driver *i*, *E*_*c*_ denoted the presence/absence of coralligenous outcrops in the grid cell, and *w*_*i*_ is the weighting coefficient that represents the impact weight score for the anthropogenic driver *i* on the focus ecosystem. Weight scores referred to those listed for “*Rocky Reefs*” in Halpern *et al*.^36^, since coralligenous outcrops can be ascribed to this ecosystem category, which include sublittoral hard bottoms up to 30–60 m depth^12^.

Finally, a regional map of estimated cumulative impact on coralligenous outcrops was obtained by assigning the corresponding expected cumulative impact to each cell of the habitat map. The same thresholds as in Halpern *et al*.^6^ and Micheli *et al*.^12^ were used to designate ecologically meaningful categories of impact scores (*I*_*c*_): very high (>15.52); high (12–15.52); medium-high (8.47–12); medium (4.95–8.47); low (1.4–4.95); and very low impact (<1.4).

### Field survey on coralligenous outcrops

Two extensive surveys along the Apulia coast were carried out in order to investigate the extent to which the estimated cumulative impact scores were aligned to the actual condition of coralligenous assemblages. Surveys were conducted in 2012 and 2013 (June-July) across a set of 26 sites. Sites were randomly selected to be representative of coralligenous outcrops from the whole region and of the range of cumulative pressures at a regional scale (Table S2 in Supplementary material), including Marine Protected Areas (MPAs), and areas featured by low, moderate and high levels of human activities. In each site, coralligenous outcrops at 20–25 m depth were photographically sampled along three 25 m-long transects (tens of meters far each other). For each transect, 8 sampling units (each one covering a surface of approximately 0.2 m^2^) were randomly selected and, for each sampling unit, a composite of 6 adjacent photographic samples (23 × 15 cm) were collected using a high-resolution digital camera. A total of 3744 photographic samples were analysed in order to estimate % cover of sessile taxa. The 64 taxa found were then grouped into four categories representing the main morpho-functional components of coralligenous outcrops (see Table S3 in Supplementary material): *Calcified Algae* (principal builders and typical component of the hard structure of coralligenous outcrops, including algae with high calcification of the thallus, e.g. encrusting corallines), *Erect Macroalgae* (contributing to bioconstruction or to three-dimensional complexity of outcrops, including algae with low calcification, e.g. *Flabellia petiolata*, *Halimeda tuna*), *Turf Algae* (opportunistic, filamentous, or turf-forming algae with low or absent calcification, e.g. *Gelidium* spp.), *Invertebrates* (typical component of outcrops, second main builders of bioconstructions after calcified algae, including sponges, bryozoans, calcareous tube worms, madreporarians and others). Since the structure of coralligenous assemblages mostly encompasses calcified algae and invertebrates^19^, whereas the dominance of turf-forming algae is a recognized indicator of disturbed conditions (e.g.^47^), shifts in dominance of these main components, from calcified algae and invertebrates towards turf-dominated assemblages, can be interpreted as a transition from healthy to degraded conditions of the outcrops.

### Statistical analyses

Thresholds of impact scores, which are generally used to classify the estimated cumulative impact in the CPIA approach, were provided in Halpern *et al*.^6^ and obtained by fitting a linear regression of the *I*_*c*_ versus the state of degradation of the system. This linear relationship was based on 16 points from different ecosystems (mostly coral reefs and associated habitats), and thresholds in *I*_*c*_ calculated to correspond to <10% (very low), 10–30% (low), 30–50% (medium), 50–70% (medium-high), 70–90% (high), and >90% (very high) percentage of ecosystem degradation. The level of degradation was based on a multivariate gradient analysis on main reef guilds, classifying ecosystems from pristine to ecologically extinct conditions^48^. Analogously, we quantified the condition of coralligenous outcrops based on their main structural components, assessing whether the linear relationship and thresholds in *I*_*c*_ derived by Halpern *et al*.^6^ applied also to our case.

First, a Principal Coordinates Analysis (PCoA) of site centroids based on Bray-Curtis similarity was carried out to visualize patterns of variation in assemblage structure among sites. The contributions of the four main components of assemblage structure to similarity patterns were visualized as correlation vectors on the ordination plot. Most of variation (>80%) in assemblage structure among sites was explained by the PCoA axis 1. Since PCoA values of sites along the axis strongly correlate with the condition of coralligenous assemblages from the best (dominance of calcified algae and animal builders) to the worse (dominance of turf-forming algae) structure (see Results), they were assumed to correspond to a gradient of increasing degradation of coralligenous outcrops. PCoA values of sites, from the lowest (best recorded condition) to the highest (worse recorded condition), along the axis 1 were rescaled to vary from 10 to 90, assuming the lowest and the highest value as the limit between very low-low and high-very high condition of degradation respectively. Then, for each of the 26 surveyed sites, values of *I*_*c*_ were plotted against the corresponding condition of assemblages obtained from the PCoA. Residuals of actual data points with respect to the expected *I*_*c*_ values from the linear model provided by Halpern *et al*.^6^ were analysed, and the Wald-Wolfowitz runs test^49^ was performed to check the correctness of the model. Runs test returns the probability that the observed pattern in the residuals is unlikely (*P* < 0.05) or not unlikely (*P* > 0.05), and therefore, that the equation used to fit data is unlike to be correct or could be correct (i.e., the residuals are randomly distributed around the fitted curve) respectively.

Other potential relationships between *I*_*c*_ and the condition of assemblages were explored by fitting linear [*y* = *ax* + *b*], logarithm [*y* = *aln(x)* + *b*], log-linear [*ln(y)* = *ax* + *b*], and log-log [*ln(y)* = *aln(x)* + *b*] models to data points. Corrected Akaike Information Criterion (AICc)^50^ was used to identify the model best fitting data, and runs tests were also performed to check patterns in the residuals. We anticipate that the log-log model performed better than any other in fitting actual data. Therefore, we re-calculated analogous thresholds in *I*_*c*_ as done by Halpern *et al*.^6^ following the log-log model. Finally, we produced a regional map of *I*_*c*_ of coralligenous outcrops based on the new thresholds and compared the results with those obtained using classic thresholds.

The relationships between the assemblage structure of sites and the full set of anthropogenic drivers was investigated using a non-parametric multivariate regression (DISTLM, distance-based multivariate multiple regression based on a linear model^51^). Prior to analysis, all explanatory variables (i.e., the variables measuring the pressure associated to anthropogenic drivers) were transformed using log(X+1) and rescaled between 0 and 1. A marginal test was employed to check the individual relationship of each driver with multivariate assemblage data and select those driver having a significant correlation with the observed patterns in coralligenous assemblages. Analyses were based on Bray-Curtis similarities and tests done using 999 permutations. All statistical analyses were carried out using R^52^.

### Data availability

Web links to all sources of pressure data and habitat maps used in the study are reported in the main text or in the Supplementary Information file. The dataset on coralligenous assemblages analysed during the current study are available from the corresponding author on reasonable request.

## Electronic supplementary material

Supplementary material
