# Supplementary material for: A regional assessment of cumulative impact mapping on Mediterranean coralligenous outcrops
Source: Sci Rep. 2018 Jan 29;8:1757. doi: 10.1038/s41598-018-20297-1 (PMC5789093; doi:10.1038/s41598-018-20297-1)

A regional assessment of cumulative impact mapping on Mediterranean coralligenous outcrops

Bevilacqua S.[1,*], Guarnieri G.[1], Farella G.[2], Terlizzi A.[3,4], Fraschetti S.[1,*]

[1]*Department of Biological and Environmental Sciences and Technologies, University of Salento, CoNISMa, 73100 Lecce, Italy*

[2]*CNR-ISMAR – Institute of Marine Sciences, Venezia, Italy*
[3]*Department of Life Sciences, University of Trieste, 34127 Trieste, Italy*
[4]*Stazione Zoologica Anton Dohrn, 80121 Napoli, Italy*

*Corresponding authors:
Stanislao Bevilacqua, e-mail: stanislao.bevilacqua@unisalento.it
Simonetta Fraschetti, e-mail: simona.fraschetti@unisalento.it

Supplementary Information

Additional material complementing the article "*A regional assessment of cumulative impact mapping on Mediterranean coralligenous outcrops*" by Bevilacqua et al. is provided in this section.

Mapping the distribution and intensity of human pressures

The full list of threats to marine ecosystems provided by Halpern et al. (2007) was taken into account in order to proceed to a full screening of potential threats to coralligenous outcrops. Fourteen threats out of a total of 35, were considered as not relevant to the habitat and geographic region under study and excluded from the analysis, along with 9 threats for which no data were available (see Table S1). The remaining 12 threats and their related drivers (Table S1), namely *Sewage discharge* (point, organic pollution), *Industrial Effluents* (point, non-organic pollution), *Agriculture* (non-point, organic pollution), *Urbanization* (non-point, non-organic pollution), *Coastal Engineering*, *Coastal Erosion* (coastal development), *Coastal Population* (direct human), *Aquaculture*, *Artisanal Fisheries* (artisanal, destructive fishing), *Ocean Acidification*, *Shipping* (ocean-based pollution), and *Commercial Activity*, were quantified and mapped in order to calculate the cumulative impact score on coralligenous outcrops (see Methods).

The intensity of pressure derived from all selected drivers (except *Artisanal Fisheries*, *Ocean Acidification*, and *Commercial Activity*, for which the potential effects were considered as acting uniformly in space and/or being confined to their area of influence) was assumed to decrease at increasing distance from the source. Negative exponential models were used to estimate the distance-decay in the intensity of pressure from 100% until 0% on a 200 m-distance raster matrix. First, vector data on each pressure driver were converted to raster, and Euclidean distances were calculated from the nearest source cell to each of the surrounding pixels until 1 to 10 km, according to Holon et al. (2015) categories. Then, the influence on marine waters of each pressure was assumed to decrease to 0% following the negative exponential equation provided by Holon et al. (2015) and based on data from monitoring programs in NW Mediterranean.

A further theoretical decrease in the intensity of pressure equal to 10% for each 10-m depth increment was applied to account for reduction of potential effects of drivers due to bathymetry (Holon et al., 2015).

All pressures were mapped on a 200 × 200 m cells grid assigning the central value to the whole cell.

A full description of pressure drivers and their use to estimate and map the distribution and intensity of selected threats is provided below.

Table S1. Selection of threats to coralligenous outcrops (within 30 m depth) based on the comprehensive list of threats to marine ecosystems provided by Halpern et al. (2007). For selected threats, the main associated human activity or socio-economic aspect (i.e. driver of pressure) is reported.

Threats to marine ecosystems	Selection	Details
Freshwater input	No	Not relevant since large sources of freshwater inputs (e.g., rivers) are absent in the study region. A few small rivers were present in the northern and western coastal sectors, where coralligenous outcrops are almost absent. The potential effects of other minor freshwater inputs (e.g., drain channels) were assumed as negligible at depths (18-30 m) and distance from the coast (> 1.5 km) characterizing the investigated portion of the habitat
Sediment input	No	No data available. However, the potential effects of other drivers (e.g., *Coastal Erosion*, *Coastal Engineering*, *Urbanization*) considered in the study are related to potential changes in sedimentation regimes through the alteration of hydrodynamism due to coastline modifications
Nutrient input	No	No data available. However, the potential effects of other drivers (e.g., *Agriculture*, *Sewage Discharge*) considered in the study are related to of organic and non-organic pollution
Pollutant input		
atmospheric	No	Not relevant for the study region since levels of atmospheric pollution at regional scale (based on annual average PM10 levels in the last 5 years) were below the thresholds fixed by law (source: www.arpa.puglia.it/web/guest/qariainq)
point organic	Yes	*Sewage discharge*
point non-organic	Yes	*Industrial effluents*
non point-organic	Yes	*Agriculture*
non point non-organic	Yes	*Urbanization*
Coastal engineering	Yes	*Coastal engineering*
Coastal development	Yes	*Coastal Erosion*
Direct human	Yes	*Coastal Population*
Aquaculture	Yes	*Aquaculture*
Fishing		
demersal, destructive	No	Not relevant for the investigated habitat since these fishing activities occur far from the coast (> 3 nautical miles) and are banned within 50 m depth. Also, this threat is of least concern due to its very low values in the study region (see Micheli et al., 2013; see also https://www.nceas.ucsb.edu/globalmarine/mediterranean)
demersal, non-destructive	No	
pelagic, high bycatch	No	Not relevant due to its very low values in the study region (see Micheli et al., 2013; see also https://www.nceas.ucsb.edu/globalmarine/mediterranean)
pelagic, low bycatch	No	
aquarium	No	No data available
illegal/unregulated/unreported	No	No data available
artisanal, destructive	Yes	*Artisanal Fisheries*
artisanal, non-destructive	No	No data available
recreational	No	No data available
Climate change		

sea level	No	Not relevant for the investigated habitat. Although an increase of 4 mm y^{-1} in sea level has been recorded in the study region (http://www.eea.europa.eu/data-and-maps), the potential effect of such changes was considered as negligible at depths characterizing the investigated habitat
sea temperature	No	Not relevant for the investigated habitat since no sea temperature anomalies were recorded in the region within the investigated depth range (see Micheli et al., 2013; see also https://www.nceas.ucsb.edu/globalmarine/mediterranean)
ocean acidification	Yes	*Ocean Acidification*
ozone/UV	No	Not relevant for the investigated habitat since the estimated 10% irradiance penetration depth for UV-A and UV-B in the Adriatic, and Mediterranean (Eastern basin excluded), seawaters was lower than 10 m (Tedetti & Sempéré, 2006).
Species invasion	No	Not relevant since very low to low rate of species invasion characterizes the region (see Micheli et al., 2013; see also https://www.nceas.ucsb.edu/globalmarine/mediterranean). The main and widespread invasive species in the region is the macroalga *Caulerpa cylindracea*, which if present was quantified as a component of assemblages during sampling activities on coralligenous outcrops (see Method section)
Disease	No	No data available
Harmful algal blooms	No	Not relevant for the investigated habitat since harmful algal blooms in the region are mostly due to *Ostreopsis ovata*, which occur very close to the coast, sporadically, and generally within 2 m depth (source: www.arpa.puglia.it/web/guest/algatossica)
Hypoxia	No	Not relevant since modelled risk of hypoxia along the region (see Micheli et al., 2013; see also https://www.nceas.ucsb.edu/globalmarine/mediterranean) was generally very low to low
Ocean-based pollution	Yes	*Shipping*
Commercial activity	Yes	*Commercial activity*
Ocean mining	No	Not relevant since absent in the study region
Offshore development	No	Not relevant because offshore extraction platforms, pipelines, and wind farms were absent in the region.
Benthic structures	No	No data available
Ecoturism	No	No data available

Sewage Discharge

Sewage discharge may introduce toxic organic compounds in the sea, which in turn could cause physiological and genetic alterations detrimental for marine organisms (Hutchinson et al*.,* 1998; Reish et al*.,* 1999; Vos et al., 2000). Also, sewage effluents could increase nutrient load, greatly affecting assemblage structure of coralligenous outcrops and bioconstruction rates (Hong 1983; Ballesteros 2006). Information on direct and indirect sewage discharge was obtained from the Register of Treatment Plants (Apulia Region decree n°1085/2009) and then georeferenced. According to Holon et al. (2015), sewage outfalls were classified into five categories based on their size in terms of population equivalent: 0) ≤4,000; 1)]4,000-10,000]; 2)]10,000-40,000]; 3)]40,000-100,000]; 4) >100,000. Distance-decay in the intensity of this driver was modelled following the equation:

1) *y=99.175e-4.56x* until 1 km for 0 or 1;

2) *y=99.175e-1.52x* until 3 for category 2;

3) *y=99.175e-0.912x* until 5 km for category 3;

4) *y=99.175e-0.456x* until 10 km category 4.

Industrial Effluents

Industrial activities are often accompanied by an increase of contaminant discharge within the natural environment. The Apulian coast hosts large industrial sites (e.g., steel factories, power plants, chemical industries). Land cover of industrial areas located within 300 m from the coastline were obtained from the Apulian Geographic Information System (SIT Puglia, http://www.sit.puglia.it) and used as a proxy of potential contamination from industrial effluents. Distance-decay in the intensity of this driver was modelled following the equation *y=99.175e-0.456x* until 10 km.

Agriculture

Massive use of pesticides and fertilizers in agricultural processes represent a potential threat to marine environment, since these compounds may reach the sea through runoff or groundwaters. While fertilizers may alter normal nutrient balance, and causing phytoplankton and algae blooms (Savage et al*.,* 2010), pesticides may have potential negative impacts on marine fauna and flora (Readman et al., 1992; Vagi et al., 2007). Land cover data of agricultural areas within 1 km from the coast were obtained from SIT Puglia (http://www.sit.puglia.it). Distance-decay in the intensity of this driver was modelled following the equation *y=99.175e-0.456x* until 10 km.

Urbanization

The replacing of natural lands by urban structures (buildings, industries, paved roads, etc.) represents a non-point source of inorganic pollution (Halpern et al*.*, 2008). Land cover data of urban areas within 1 km from the coast were extracted from SIT Puglia (http://www.sit.puglia.it) and used as a proxy of potential contamination due to urban runoffs. Distance-decay in the intensity of this driver was modelled following the equation *y=99.175e-0.456x* until 10 km.

Coastal Engineering

Coastal engineering represents reclamation from the sea (e.g., harbours, ports, groins, pontoons) causing irreversible destructions of sublittoral seabed, causing important changes in the hydrodynamics and siltation around these structures. Size and position of coastal artificial structures were obtained from SIT Puglia (http://www.sit.puglia.it). Coastal structures were then classified in three categories according to Holon et al. (2015): 1) harbours, 2) ports of refuge, 3) pontoons. Distance-decay in the intensity of this driver was modelled following the equation:

1) *y=99.175e-0.456x* until 10 km for category 1;

2) *y=99.175e-1.52x* until 3 km for category 2;

3) *y=99.175e-0.912x* until 1 km for category 3.

Coastal Erosion

Coastal erosion relates to anthropogenic pressures due to intensive use and alteration of coastal environments (Micheli et al., 2013, Holon et al., 2015). Data on the Apulian coastlines subject to critical erosion trends where gathered from SIT Puglia (http://www.sit.puglia.it). Distance-decay in the effects of this driver was modelled following the equation to the equation: *y=99.175e-1.52x* until 3 km.

Coastal Population

The Apulian coast is densely populated (up to 3,000 inhabitants per km of coast) and characterized by urban settlements and facilities. Population along the coast determines an increased need in resources (water, energy, raw material) and the emission of various discards in waters, soils and air (Savage et al., 2010). A GIS layer on population size and density of coastal municipalities (ISTAT, 2013; dati.istat.it) was built. Demographic data were ranked as follows:

- Population size: (0) ≤500, (1)]500;2,000], (2)]2,000;15,000], (3)]15,000;50,000], (4)]50,000;200,000], (5) >200,000 inhabitants;
- Population density: (0) ≤10, (1)]10;30], (2)]30;80], (3)]80;300], (4)]300;2,000], (5) >2,000 inhabitants/km^2;

A global rank, equal to the mean between ranks of population size and density, was assigned to each municipality. Distance-decay in the intensity of this driver was modelled following the equation:

1) *y=99.175e-4.56x* until 1 km for a global rank of 0, 1 or 2;

2) *y=99.175e-1.52x* until 3 km for a global rank of 3;

3) *y=99.175e-0.912x* until 5 km for a global rank of 4;

4) *y=99.175e-0.228x* until 20 km for a global rank of 5.

Aquaculture

Aquaculture can affect marine systems in different ways by introducing alien species or pathogens, increasing organic load or chemical contamination, and causing genetic degradation of wild populations (IUCN, 2007). Among the potential impacts, the organic enrichment of water, particularly relevant for fish cages farms at sea, can significantly alter the balance of nitrogen and phosphorus in the marine environment. Several studies show that excess in organic matters and nutrients from aquaculture decreases the available luminosity by eutrophication (Porrello et al., 2005), with potential formation of sulphides and sedimentary hypoxia (Holmer et al., 2008) and accumulation of organic matters within the sediment (Delgado et al., 1999). For the benthic macrofauna, impact is generally noticed until about 1 km, although this distance could vary depending on sediment grain (Apostolaki et al., 2007). Georeferenced data on the extension of aquaculture farms where gathered from SIT Puglia (http://www.sit.puglia.it). Distance-decay in the effects of this driver was modelled following the equation:

1) $y=99.175e-9.119x$ until 500 m for small farms ($\leq$4,000 m^2);

2) $y=99.175e-4.56x$ until 1 km for big farms (> 4,000 m^2).

Artisanal Fisheries

Fishery activities are a well-known cause of coralligenous degradation (Ballesteros, 2006). Overexploitation using destructive gears physically damage the coralligenous structure, while the effects of overfishing on coastal areas may cause alterations of trophic webs (Steneck, 1998). We used data from EU's Fleet Register (http://ec.europa.eu/fisheries/fleet/index.cfm) to create a probability map of artisanal fishing pressure around ports (fishery points), up to 50 m depth. Specifically, we considered vessels using bottom gillnets, which can be considered the most harmful gears for coralligenous outcrops. For each fishery point, according to Colloca et al. (2004), fishing vessels where classified per gross register tonnage (GRT) and vessel length in "coastal exploiters" (i.e., vessels ≤6 GRT and ≤7 m length) and "offshore exploiters" (i.e., vessels >7 GRT and >7 m

length), taking into account that the latter extend their fishing activities over a larger area with respect to the former. Therefore, two separate buffers were built around each fishery point: a 3 km and 10 km-radius buffer for coastal and offshore exploiters respectively, assuming that the intensity of fisheries (quantified as the number of vessels) is acting uniformly within the buffers. In case of overlapping, the two buffers were merged using ArcGIS, and summing the number of vessels at the intersections.

Ocean Acidification

The ability of calcifying species such as corals and shelled invertebrates to create calcium carbonate structures is strongly affected by ocean acidification (Kleypas et al., 1999). The reduction in the aragonite saturation state (ASS) of sea from pre-industrial (~1870) to modern times (2000-2009), was used as estimate of the human-driven changes in ocean acidification. Data were extracted from the global distribution of ASS values modelled at 1-degree resolution provided by Halpern et al. (2008).

Shipping

Pollution derived from shipping (e.g., leaks in loading/unloading operations, residues of cargos and storage, contaminated surfaces, ballast waters, invasive species introduction, antifouling procedures) could exert detrimental effects on marine systems (Gomez et al., 2015). Based on the approach of Halpern et al. (2008), a GIS layer of main commercial traffic route lanes and cargo areas (www.marinetraffic.com) was built to localize coastal areas of intense shipping. The total tonnage of shipped goods (2013) for each area (http://www.assoporti.it/statistiche/annuali) was then used as a proxy of the intensity of pollution related to this activity. Distance-decay in the intensity of this driver was modelled following the equation $y=99.175e-0.456x$ until 10 km.

Commercial Activity

Commercial maritime traffic data may provide an estimate of the occurrence of ships at a particular location, and therefore an estimate of the amount of pollution they may produce (fuel leaks, oil discharge, waste disposal). According to Halpern et al. (2008), a GIS layer of main maritime route lanes (www.marinetraffic.com) was built to identify areas of heavy maritime traffic and the intensity of this driver, expressed as the number of ship tracks per cell (200 × 200 m). We assumed that pollutants are likely to be most concentrated in traffic areas and that traveling ships primarily affect their immediate waters (Halpern et al., 2008) and, therefore, no buffer areas were applied.

References

Apostolaki, E.T., Tsagaraki, T., Tsapaki, M. & Karakassis, I. (2007). Fish farming impact on sediments and macrofauna associated with seagrass meadows in the Mediterranean. Estuarine Coastal and Shelf Science 75, 408–416. doi: 10.1016/j.ecss.2007.05.024

Ballesteros, E. (2006). Mediterranean coralligenous assemblages: a synthesis of present knowledge. Oceanography and Marine Biology: An Annual Review, 44, 123–195. doi: 10.1201/9781420006391.ch4

Colloca, F., Crespi, V., Cerasi, S., & Coppola, S.R. (2004). Structure and evolution of the artisanal fishery in a southern Italian coastal area. Fisheries Research, 69, 359–369. doi: 10.1016/j.fishres.2004.06.014

Delgado, O., Ruiz, J., Pérez, M., Romero, J. & Ballesteros, E. (1999). Effects of fish farming on seagrass (*Posidonia oceanica*) in a Mediterranean bay: seagrass decline after organic loading cessation. Oceanologica Acta, 22, 109–117. doi: 10.1016/S0399-1784(99)80037-1

Gomez, A. G., Ondiviela, B., Puente, A., & Juanes J. A. (2015). Environmental risk assessment of water quality in harbor areas: A new methodology applied to European ports. Journal of Environmental Management 155, 77–88. doi: 10.1016/j.jenvman.2015.01.042

Halpern, B. S., Walbridge, S., Selkoe, K. A., Kappel, C. V., Micheli, F., D'Agrosa, C., … Watson, R. (2008). A global map of human impact on marine ecosystems. Science, 319, 948–952. doi: 10.1126/science.1149345

Holon, F., Mouquet, N., Boissery, P., Bouchoucha, M., Delaruelle, G., Tribot, A. S., & Deter J. (2015). Fine-Scale Cartography of Human Impacts along French Mediterranean Coasts: A Relevant Map for the Management of Marine Ecosystems. PLoS ONE, 10(8), e0135473. doi: 10.1371/journal.pone.0135473

Hong, J. S. (1983). Impact of the pollution on the benthic community: environmental impact of the pollution on the benthic coralligenous community in the Gulf of Fos, northwestern Mediterranean. Bulletin of the Korean Fisheries Society, 16, 273–290.

Holmer, M., Black, K., Duarte, C. M., Marbà, N., & Karakasis, I. (2008). *Aquaculture in the Ecosystem*. Springer

Hutchinson, T. H., Awadhesh, N. S., MacKay, J. M., Elliott, B. M., & Dixon, D. R. (1998). Assessment of developmental effects, cytotoxicity and genotoxicity in the marine polychaete (*Platinereis dumerilii*) exposed to disinfected municipal sewage effluent. Mutation Research, 399, 97–108.

Kleypas, J. A., Buddemeier, R. W., Archer, D., Gattuso, J. P., Langdon, C., & Opdyke, B. N. (1999). Geochemical Consequences of Increased Atmospheric Carbon Dioxide on Coral Reefs. Science, 284 (5411), 118–120. doi: 10.1126/science.284.5411.118

Micheli, F., Halpern, B. S., Walbridge, S., Ciriaco, S., Ferretti, F., Fraschetti, F., … Rosenberg, A. (2013). Cumulative Human Impacts on Mediterranean and Black Sea Marine Ecosystems: Assessing Current Pressures and Opportunities. PLoS One, 8(12), e79889. doi: 10.1371/journal.pone.0079889

Porrello, S., Tomassetti, P., Manzueto, L., Finoia, M. G., Persia, E., Mercatali, I. & Stipa, P. (2005). The influence of marine cages on the sediment chemistry in the western Mediterranean Sea. Aquaculture, 249: 145−158. doi: 10.1016/j.aquaculture.2005.02.042

Readman, J.W., Kwong, L. L. W., Mee, l. D., Bartocci, J., Nilve, G., & Rodriguez-Solano, J. A., Gonzalez-Farias, F. (1992). Persistent organophosphorus pesticides in tropical marine environments. Marine Pollution Bulletin 24 (8), 398–402. doi: 10.1016/0025-326X(92)90500-6

Reish, D. J., Oshida, P. S., Mearns, A. J., Ginn, T. C., & Buchman, M. (1999). Effects of pollution on marine organisms. Water Environment Research, 71, 1100–1115.

Savage, C., Leavitt, P. R., & Elmgren, R. (2010). Effects of land use, urbanization, and climate variability on coastal eutrophication in the Baltic Sea. Limnology and Oceanography, 55(3), 1033–1046. doi:10.4319/lo.2010.55.3.1033

Steneck, R.S. (1998). Human influences on coastal ecosystems: Does overfishing create trophic cascades? Trends in Ecology and Evolution, 13, 429430.

Vagi, M. C., Petsas, A. S., Kostopoulou, M. N., Karamanoli, M. K., & Lekkas, T.D. (2007). Determination of organochlorine pesticides in marine sediments samples using ultrasonic solvent extraction followed by GC/ECD. Desalination, 210, 146–156. doi: 10.1016/j.desal.2006.06.020

Vos, J. G., Dybing, E., Greim, H. A., Ladefoged, O., Lambre, C., Tarazona, J. V., … Vethaak, A. D. (2000). Health-Effects of endocrine-disrupting chemicals on wildlife, with special reference to the European situation. Critical Reviews in Toxicology, 30, 71–133. doi: 10.1080/10408440091159176

Regional maps of drivers

In this section, we provided maps for pressures' layers at a regional scale. A map of *Ocean Acidification* is omitted since this data layer at Mediterranean scale is available at http://globalmarine.nceas.ucsb.edu/mediterranean (Micheli et al., 2013). Also, maps of *Coastal Erosion*, *Aquaculture*, and *Commercial Activity* were not reported because the overlay analysis of spatial distribution of these drivers excluded any potential interference with coralligenous outcrops within 30 m depth. Images were colour-ramped by rescaling driver values between 0 and 100%, where 100% corresponded to the maximum value recorded in the region. Maps in figures S1, S2, S3, S4, S5, S6, S7, S8 were created using the ArcGIS® 10.1 software by ESRI (Environmental Systems Resource Institute, http://www.esri.com).

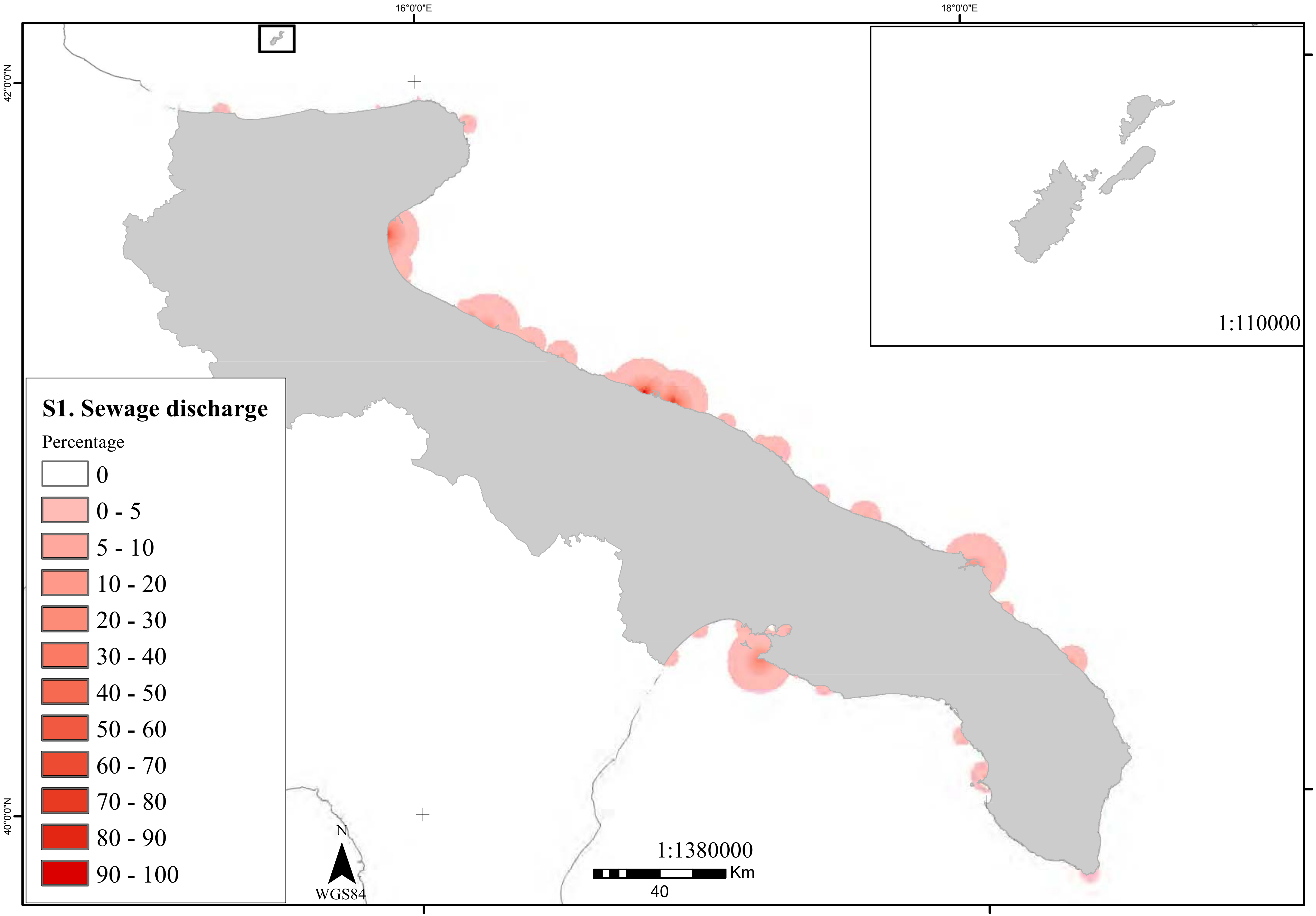
16°0'0"E
18°0'0"E
42°0'0"N
40°0'0"N
1:110000
S1. Sewage discharge
Percentage
0
0 - 5
5 - 10
10 - 20
20 - 30
30 - 40
40 - 50
50 - 60
60 - 70
70 - 80
80 - 90
90 - 100
N
WGS84
1:1380000
Km
40

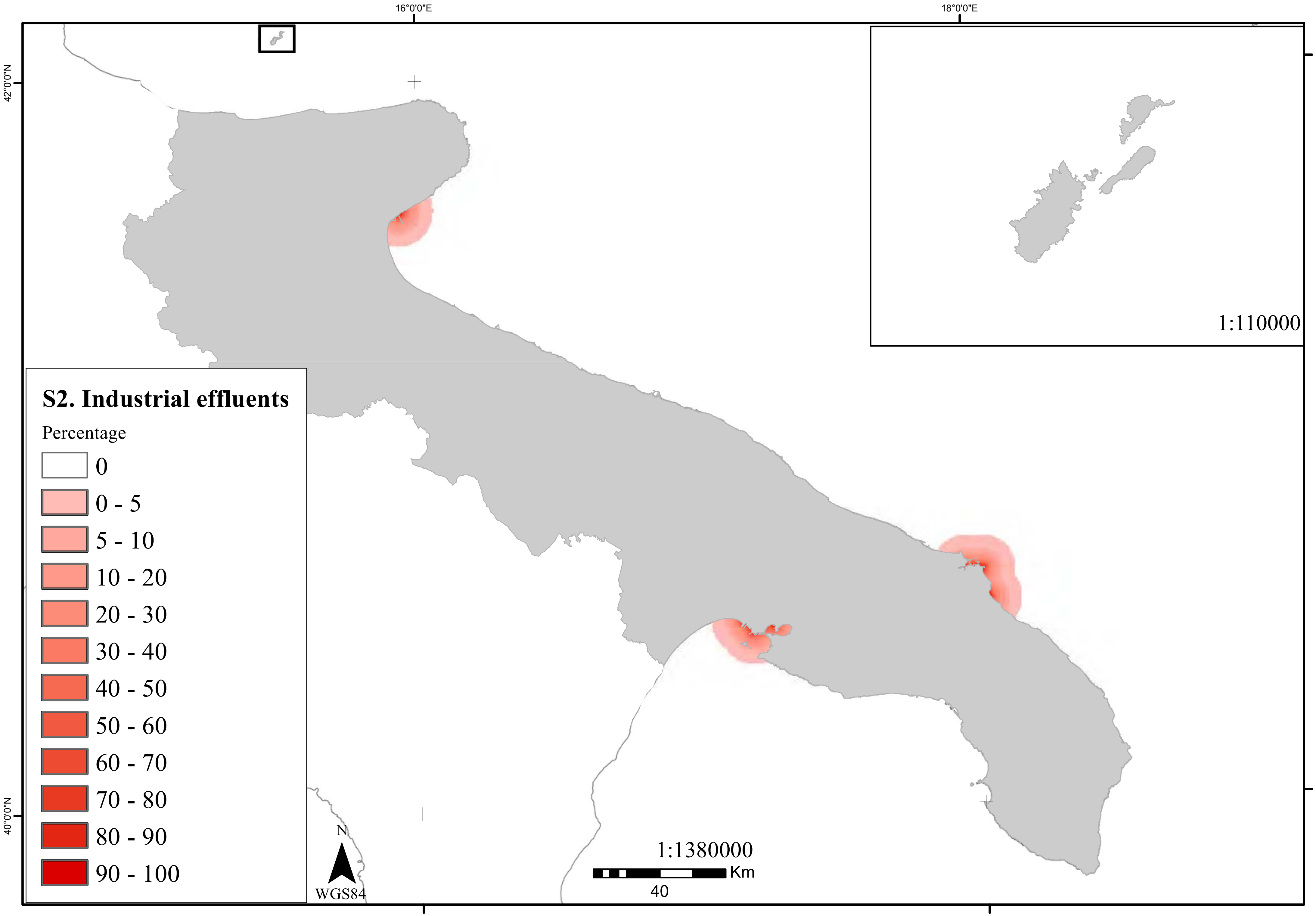
16°0'0"E
18°0'0"E
42°0'0"N
40°0'0"N
1:110000
S2. Industrial effluents
Percentage
0
0 - 5
5 - 10
10 - 20
20 - 30
30 - 40
40 - 50
50 - 60
60 - 70
70 - 80
80 - 90
90 - 100
N
WGS84
1:1380000
Km
40

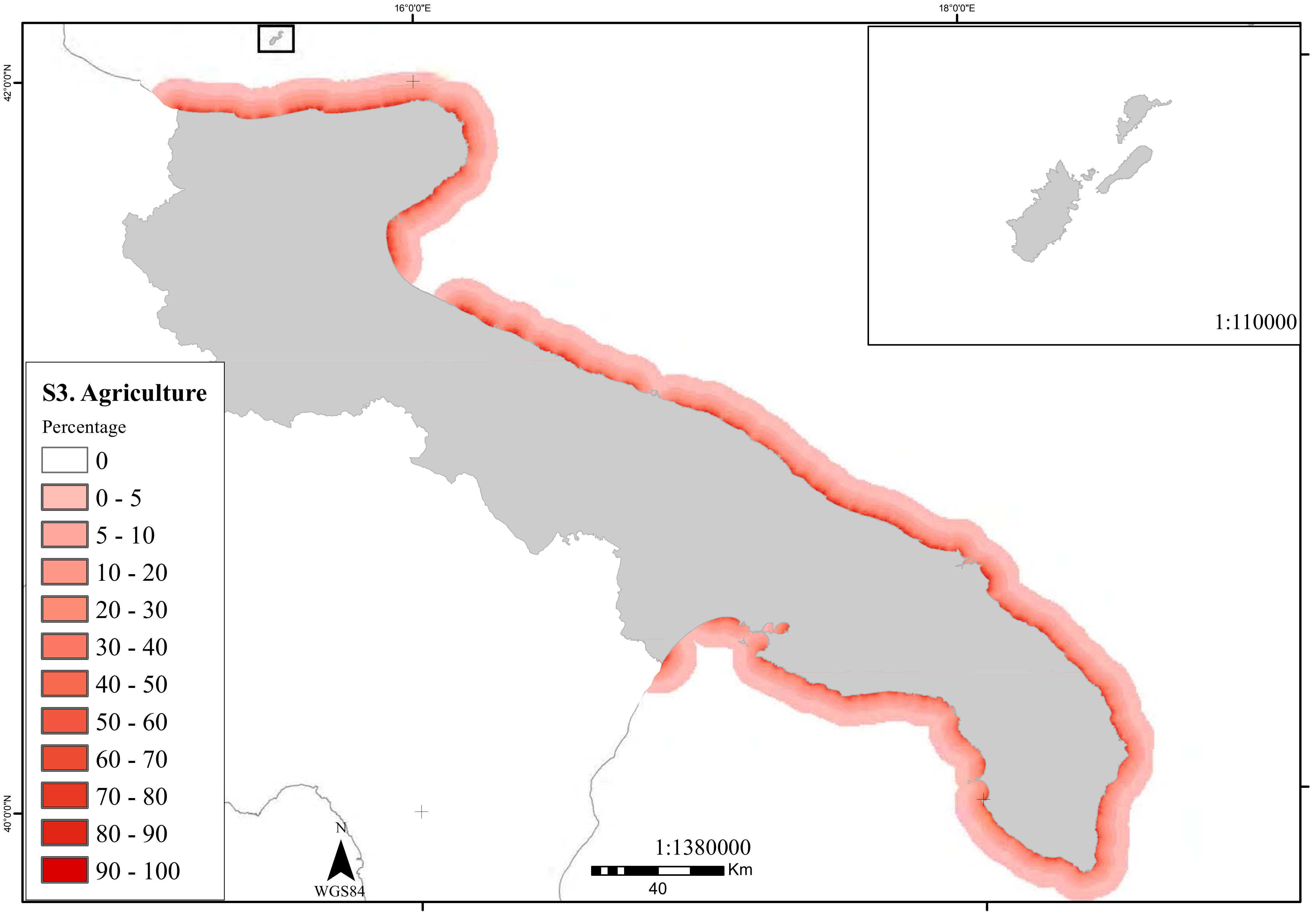
16°0'0"E
18°0'0"E
42°0'0"N
40°0'0"N
1:110000
S3. Agriculture
Percentage
0
0 - 5
5 - 10
10 - 20
20 - 30
30 - 40
40 - 50
50 - 60
60 - 70
70 - 80
80 - 90
90 - 100
N
WGS84
1:1380000
Km
40

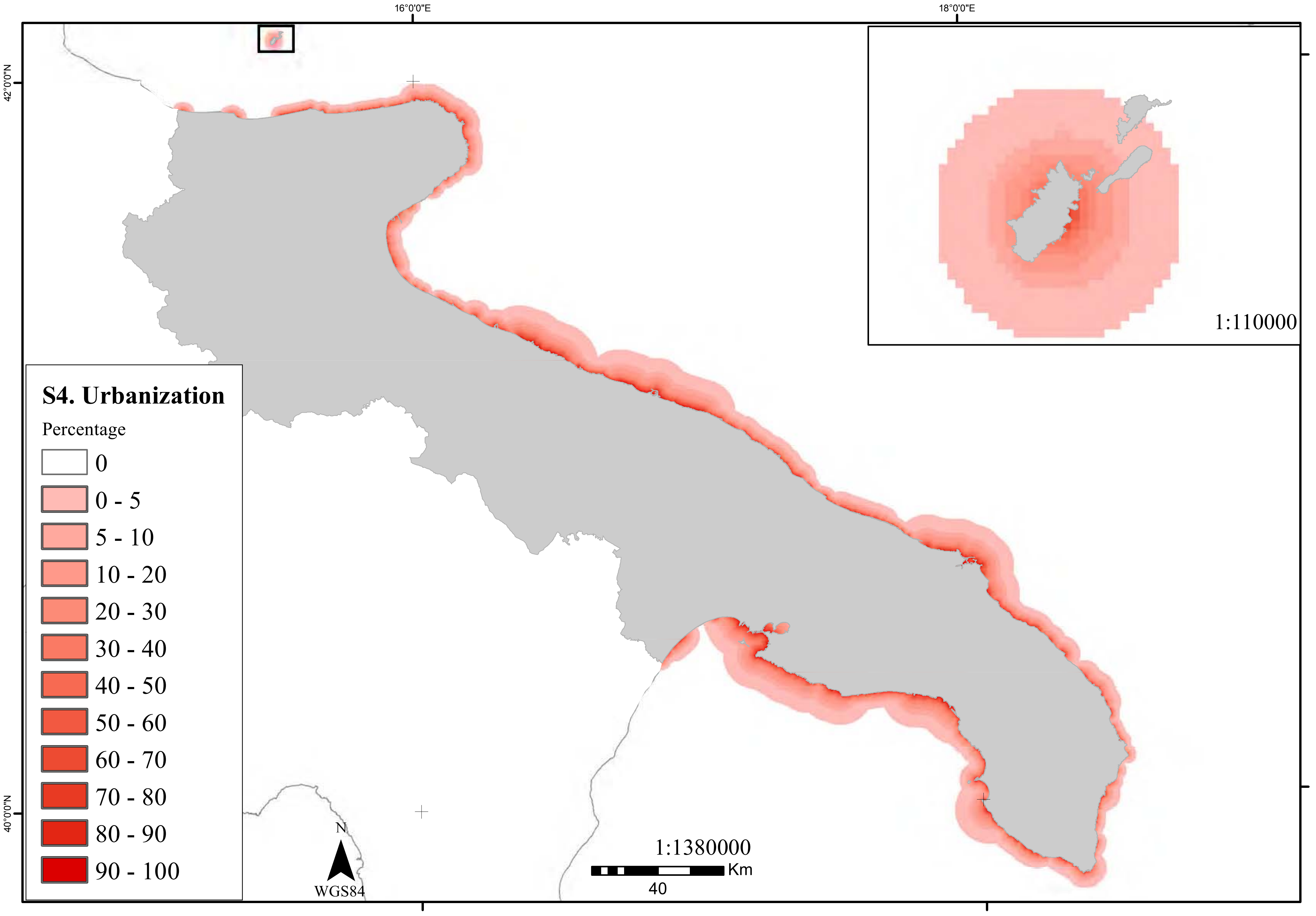
16°0'0"E
18°0'0"E
42°0'0"N
40°0'0"N
1:110000
S4. Urbanization
Percentage
0
0 - 5
5 - 10
10 - 20
20 - 30
30 - 40
40 - 50
50 - 60
60 - 70
70 - 80
80 - 90
90 - 100
N
WGS84
1:1380000
Km
40

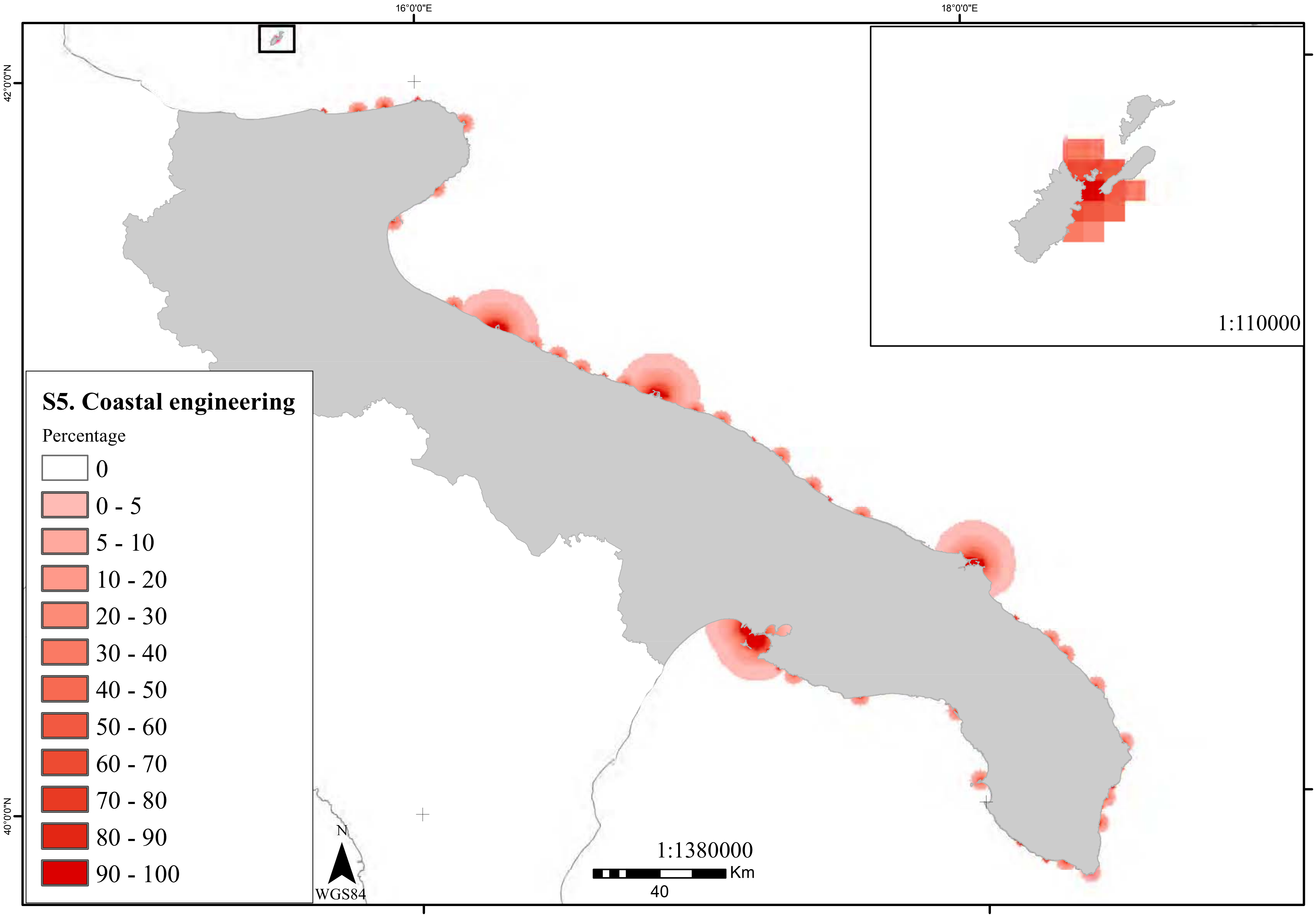
16°0'0"E
18°0'0"E
42°0'0"N
40°0'0"N
1:110000
S5. Coastal engineering
Percentage
0
0 - 5
5 - 10
10 - 20
20 - 30
30 - 40
40 - 50
50 - 60
60 - 70
70 - 80
80 - 90
90 - 100
N
WGS84
1:1380000
Km
40

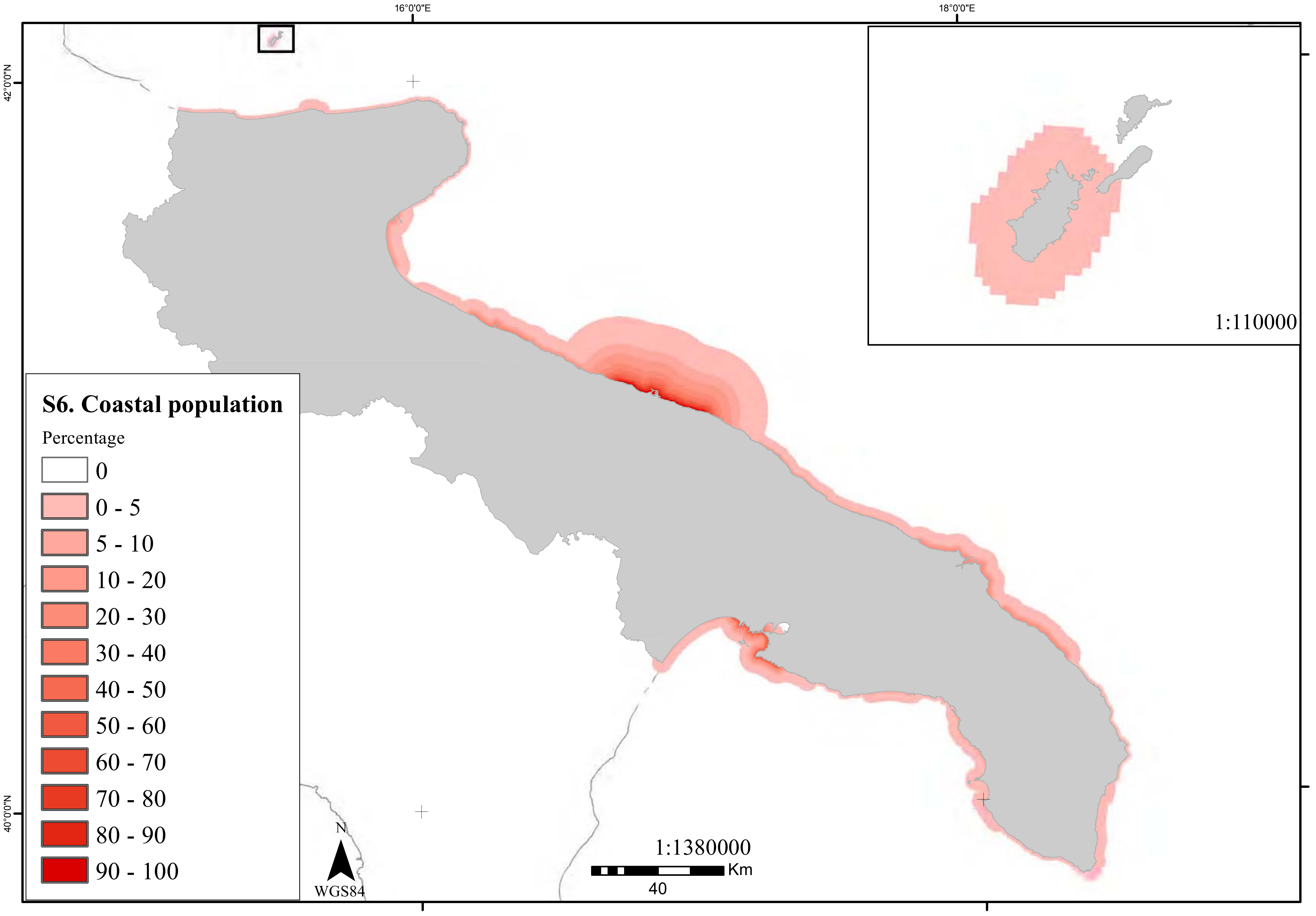
16°0'0"E
18°0'0"E
42°0'0"N
40°0'0"N
1:110000
S6. Coastal population
Percentage
0
0 - 5
5 - 10
10 - 20
20 - 30
30 - 40
40 - 50
50 - 60
60 - 70
70 - 80
80 - 90
90 - 100
N
WGS84
1:1380000
Km
40

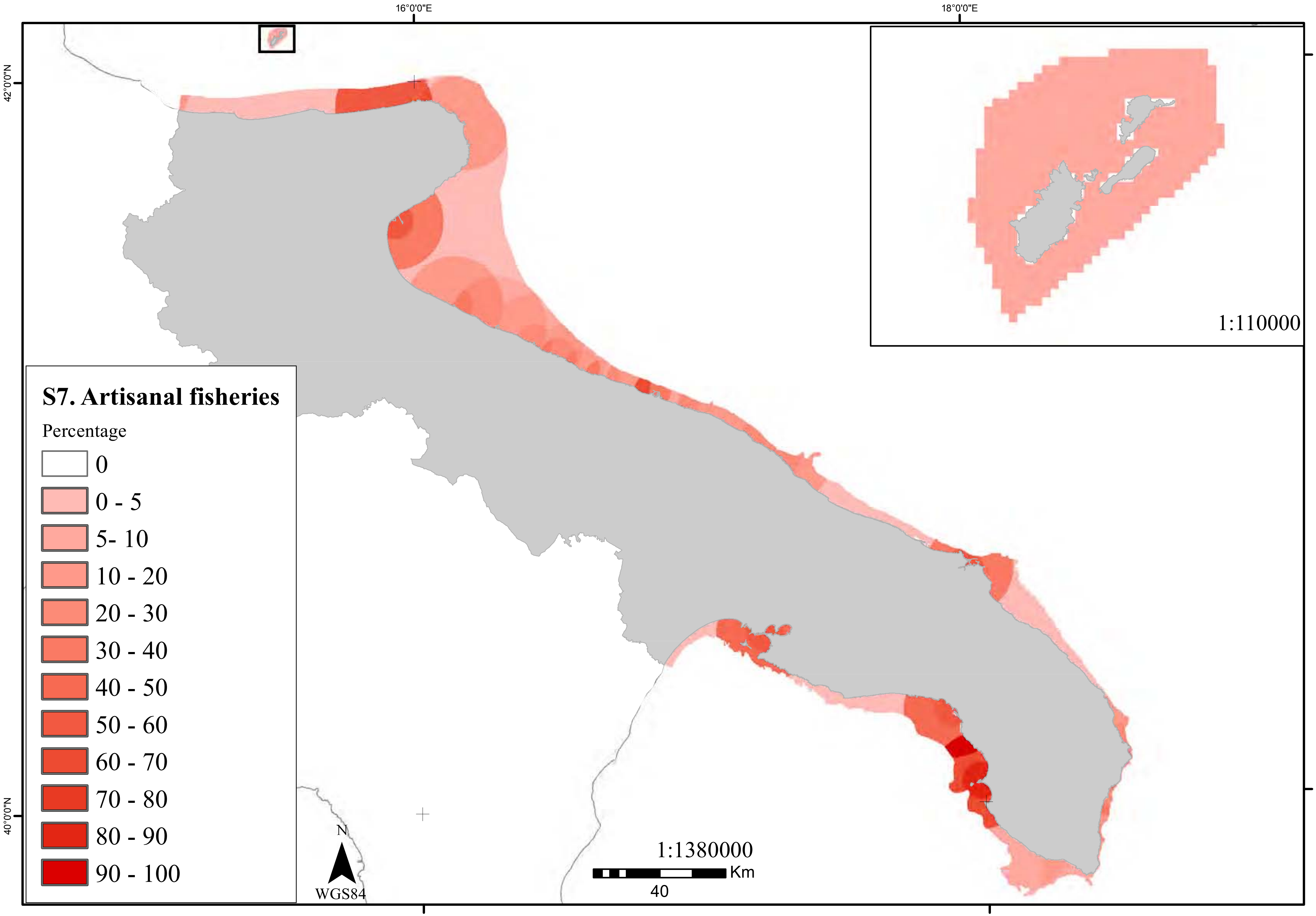

16°0'0"E
18°0'0"E
42°0'0"N
40°0'0"N
1:110000
S7. Artisanal fisheries
Percentage
0
0 - 5
5- 10
10 - 20
20 - 30
30 - 40
40 - 50
50 - 60
60 - 70
70 - 80
80 - 90
90 - 100
N
WGS84
1:1380000
Km
40

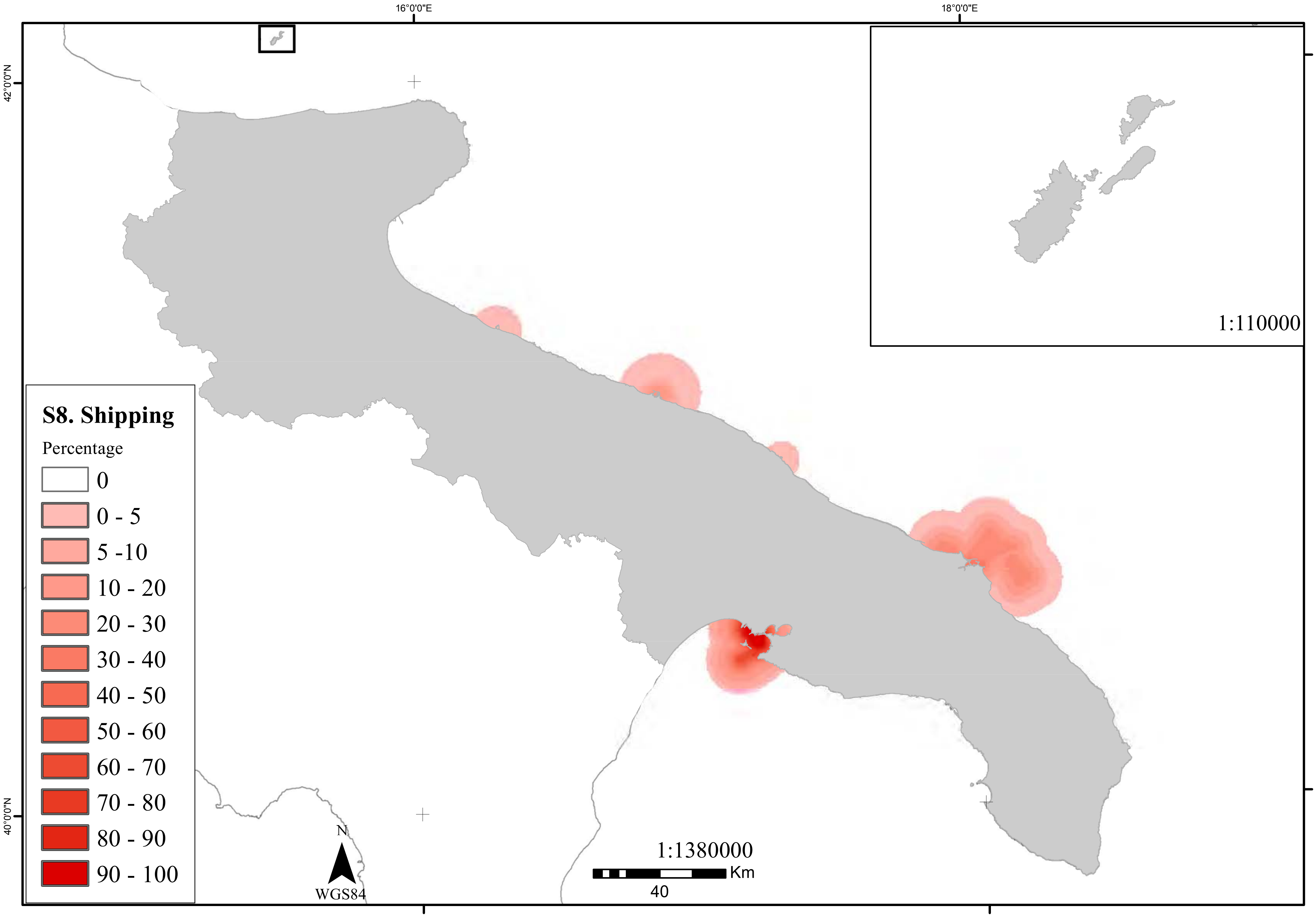
16°0'0"E
18°0'0"E
42°0'0"N
40°0'0"N
1:110000
S8. Shipping
Percentage
0
0 - 5
5 -10
10 - 20
20 - 30
30 - 40
40 - 50
50 - 60
60 - 70
70 - 80
80 - 90
90 - 100
N
WGS84
1:1380000
Km
40

Table S2. Normalized values (*D*) of each pressure in sampled sites. The original values were log (X+1) transformed and rescaled to vary between 0-1 (Micheli et al., 2013) (see text for details). Classes of intensity, from 1.00-0.8 to ≥ 0.2 respectively, were highlighted with colours ranging from dark to light grey.

Site	*Lat*	*Long*	*Ocean Acidification*	*Artisanal Fisheries*	*Urbanization*	*Agriculture*	*Coastal Population*	*Coastal Engineering*	*Sewage Discharge*	*Shipping*	*Industrial Effluents*	$\sum D_i$
1	42°08'21.91"N	15°31'08.94"E	1.00	0.46	0.00	0.00	0.00	0.00	0.00	0.00	0.00	1.46
2	42°07'23.43"N	15°29'06.66"E	1.00	0.46	0.52	0.00	0.28	0.00	0.00	0.00	0.00	2.27
3	42°07'15.69"N	15°30'43.42"E	1.00	0.46	0.28	0.00	0.00	0.73	0.00	0.00	0.00	2.47
4	40°15'46.00"N	17°51'21.00"E	1.00	0.89	0.76	0.63	0.00	0.00	0.00	0.00	0.00	3.27
5	40°44'20.00"N	17°46'35.00"E	1.00	0.34	0.32	0.78	0.55	0.00	0.00	0.47	0.00	3.45
6	39°49'33.00"N	18°23'29.00"E	1.00	0.59	0.37	0.92	0.63	0.00	0.00	0.00	0.00	3.50
7	40°03'07.00"N	18°28'40.00"E	1.00	0.70	0.40	0.80	0.58	0.00	0.37	0.00	0.00	3.84
8	39°57'59.00"N	17°59'47.00"E	1.00	0.93	0.76	0.63	0.55	0.00	0.00	0.00	0.00	3.87
9	39°49'15.00"N	18°16'47.00"E	1.00	0.55	0.76	0.75	0.32	0.73	0.00	0.00	0.00	4.10
10	40°01'42.00"N	18°26'37.00"E	1.00	0.00	0.83	0.89	0.58	0.89	0.00	0.00	0.00	4.20
11	40°11'47.00"N	17°54'13.00"E	1.00	0.85	0.51	0.75	0.65	0.00	0.44	0.00	0.00	4.21
12	40°15'01.00"N	17°53'12.00"E	1.00	0.89	0.86	0.62	0.44	0.54	0.00	0.00	0.00	4.35
13	39°47'18.00"N	18°21'27.00"E	1.00	0.55	0.79	0.75	0.46	0.70	0.57	0.00	0.00	4.80
14	40°09'52.00"N	17°56'45.00"E	1.00	1.00	0.85	0.82	0.78	0.00	0.39	0.00	0.00	4.83
15	39°47'31.00"N	18°22'06.93"E	1.00	0.55	0.77	0.72	0.60	0.76	0.55	0.00	0.00	4.94
16	40°59'42.98"N	17°14'13.44"E	1.00	0.76	0.84	0.94	0.77	0.00	0.73	0.00	0.00	5.05
17	40°26'23.00"N	17°08'46.00"E	1.00	0.83	0.33	0.17	0.54	0.66	0.69	0.88	0.28	5.37
18	40°58'30.84"N	17°16'45.12"E	1.00	0.75	0.84	0.83	0.76	0.34	0.75	0.17	0.00	5.45
19	40°28'11.00"N	17°07'42.00"E	1.00	0.83	0.63	0.37	0.72	0.75	0.00	0.86	0.44	5.59
20	40°57'27.42"N	17°18'34.16"E	1.00	0.75	0.81	0.78	0.78	0.86	0.60	0.37	0.00	5.97
21	41°07'06.83"N	16°56'11.59"E	1.00	0.57	0.84	0.75	0.97	0.54	0.93	0.56	0.00	6.15
22	40°23'37.00"N	17°13'49.00"E	1.00	0.83	0.82	0.85	0.87	0.35	0.88	0.79	0.00	6.39
23	40°24'06.16"N	17°11'58.18"E	1.00	0.83	0.84	0.61	0.88	0.51	0.89	0.85	0.07	6.48
24	41°08'08.16"N	16°53'32.41"E	1.00	0.82	0.81	0.37	0.96	0.95	0.86	0.71	0.00	6.49
25	41°09'16.74"N	16°49'02.84"E	1.00	0.92	0.79	0.64	0.96	0.75	0.92	0.60	0.00	6.59
26	40°40'05.00"N	17°58'51.00"E	1.00	0.88	0.87	0.59	0.76	0.97	0.79	0.81	0.65	7.31

Table S3. List of taxa recorded and aggregation in main morpho-functional components of coralligenous outcrops.

TAXONOMIC GROUP	TAXON	MORFO-FUNCTIONAL COMPONENT	DESCRIPTION
ALGAE	*Peyssonnelia* spp.<br>*Tricleocarpa fragilis*<br>Encrusting Calcified Rhodophytes	CALCIFIED ALGAE	Algae with high to very high calcification of the thallus. Encrusting or prostrate. Principal builders and typical component of the hard structure of coralligenous outcrops, and other bioconstructions in the Mediterranean Sea
	Chrysymenia ventricosa<br>*Codium* spp.<br>Cutleriales<br>Dumontiaceae<br>*Flabellia petiolata*<br>*Halimeda tuna*<br>*Laurencia* spp.<br>*Palmophyllum crassum*<br>*Sphaerococcus coronopifolius*<br>*Wrangelia* spp.	ERECT MACROALGAE	Algae with low or absent calcification of the thallus. Erect (e.g., *Laurencia* spp.) or massive (e.g., *Codium bursa*). Some species could partially contribute to bioconstruction (e.g., *Halimeda tuna*), or participating to form the three-dimensional structure of coralligenous assemblages with their canopy (e.g., *Sphaerococcus coronopipholius*)
	Acetabularia acetabulum<br>Articulated Corallines<br>*Caulerpa cylindracea*<br>*Colpomenia sinuosa*<br>Dark Filamentous Algae<br>Dictyotales<br>*Gelidium* spp.<br>Green Filamentous Algae<br>*Padina pavonica*<br>*Valonia macrophysa*	TURF ALGAE	Algae with low or absent calcification of the thallus. Turf-forming species. This category includes opportunistic species (e.g., Green Filamentous Algae), species that could indicate organic enrichment (e.g., *Colpomenia sinuosa*, *Gelidium* spp.), species not typical of this habitat (e.g., *Padina pavonica*), or turf-forming alien species (e.g., *Caulerpa cylindracea*). This component generally increases at increasing degradation of environmental conditions.
ANNELIDA	Filograna sp.<br>Serpulidae	INVERTEBRATES	In most cases these organisms exhibit high to very high mineralization, due to shells (e.g., molluscs),

BRYOZOA	Encrusting bryozoans	spicules (sponges), and calcified exo- or endoskeletons (e.g., bryozoans, madreporarians). Erect, encrusting or massive forms, which strongly contribute to determine the three-dimensional structure of coralligenous assemblages. Typical component of the hard structure of coralligenous outcrops, and main builders of bioconstructions besides calcified algae.
	Myriapora truncata	
	Pentapora fascialis	
	Sertella spp.	
	Thin ramified bryozoans	
CNIDARIA	*Aglaeophenia* sp.	
	Aiptasia mutabilis	
	Balanophyllia europaea	
	Caryophyllia spp.	
	Cerianthus membranaceus	
	Cladocora caespitosa	
	Eunicella spp.	
	Hydrozoans	
	Leptopsammia pruvoti	
	Parazoanthus axinellae	
MOLLUSCA	*Gastrochaena dubia*	
	Vermetidae	
PORIFERA	*Acanthella acuta*	
	Agelas oroides	
	Axinella spp.	
	Chondrilla nucula	
	Chondrosia reniformis	
	Clathrina clathrus	
	Cliona spp.	
	Dysidea avara	
	Encrusting red sponges	
	Haliclona mediterranea	
	Hemimycale columella	
	Hexadella racovitzai	
	Massive dark sponges	
	Massive sponges	
	Oscarella lobularis	

	Petrosia ficiformis
	Phorbas spp.
	Terpios fugax
TUNICATA	*Aplydium* spp.<br>*Cystodytes dellechiajei*<br>Didemnidae<br>*Halocynthia papillosa*

Figure S9. Principal Coordinates Analysis (PCoA) of site centroids based on Bray-Curtis similarity among coralligenous assemblages. Assemblage structure was defined based on four main components of coralligenous outcrops, and namely *Calcified algae*, *Erect macroalgae*, *Turf algae*, and *Invertebrates* (see Table S3). Correlation vectors of assemblage components were visualized in the ordination plot. Numbers indicated sites as in Table S2.

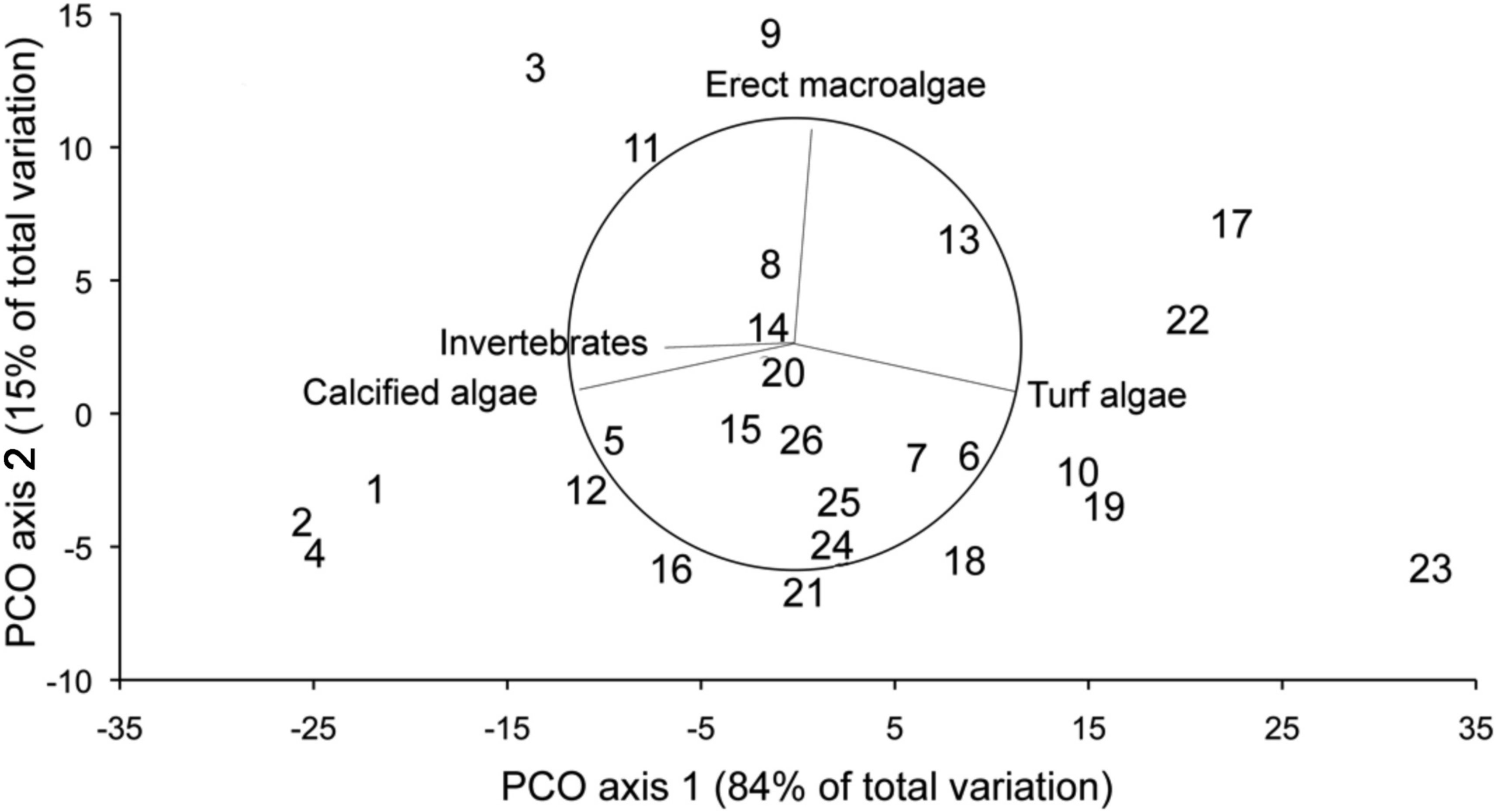

Figure S10. Plot of residuals of I_c from actual data and predicted I_c from the linear relationships provided by Halpern et al. (2008) against the independent variable (condition of the assemblage). Inspection of plot showed residuals to be biased by an upward to downward trend (red line), indicating that, in our case, the linear function of Halpern et al. (2008) did not explain adequately the relationship between I_c and the condition of assemblages.

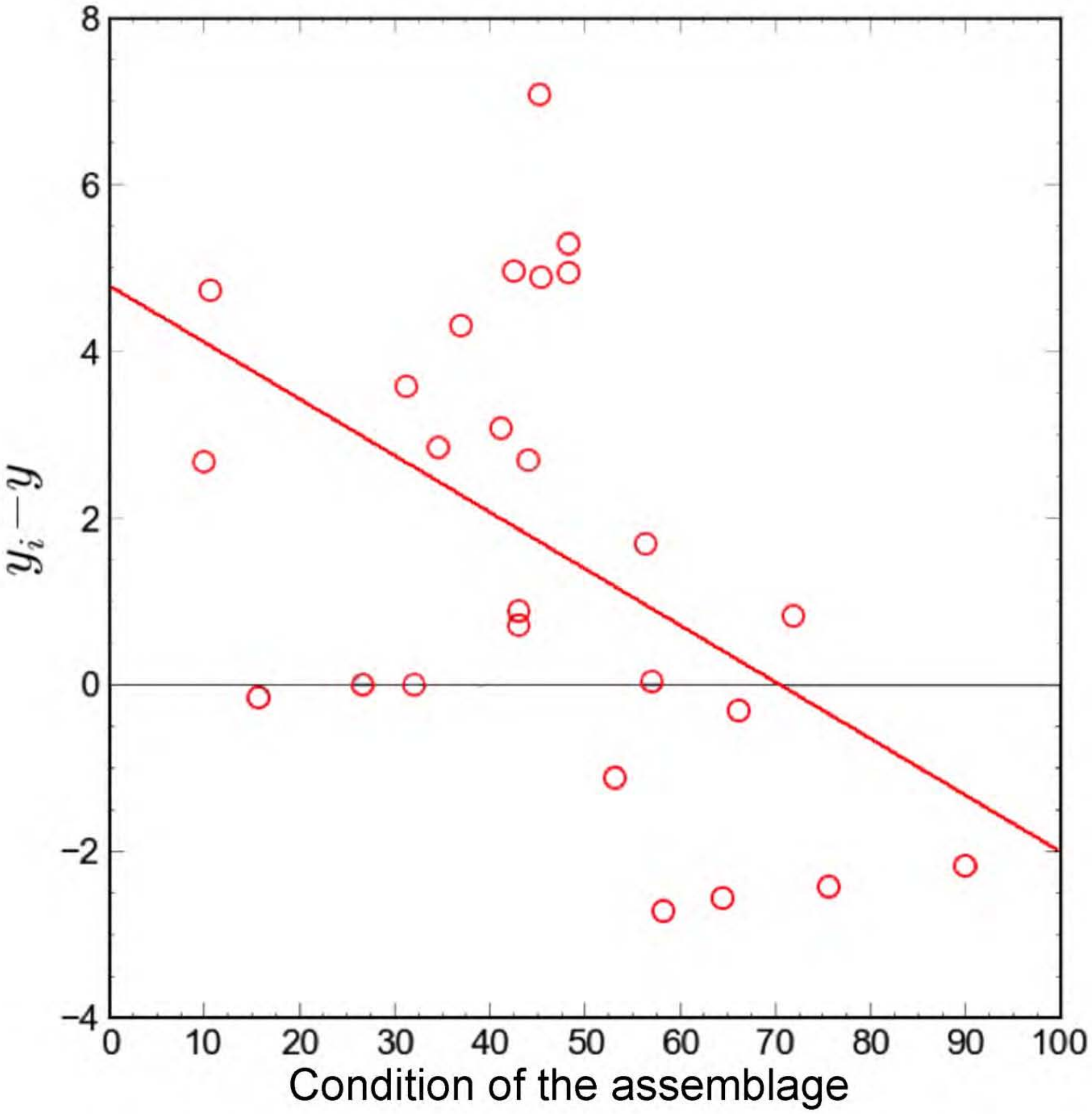

Supplement: Supplementary file 1 — Supplementary material [file 41598_2018_20297_MOESM1_ESM.pdf]
